# Roadmap for the implementation of particle minibeam therapy

**DOI:** 10.1002/mp.70660

**Published:** 2026-09-14

**Authors:** Reem Ahmad, Oxana Actis, Adam H. Aitkenhead, Richard A. Amos, Niels Bassler, Catherine Burne, Pankaj Chaudhary, Gerd Datzmann, Umberto Deut, Fabio Di Martino, Rebecca Habgood, Simon Jolly, Emmanuel Jouglar, Eva Kasanda, Nigel Lee, Dominic Leiser, Xiangkun Li, Giuliana Milluzzo, Jessica Neubauer, Kelvin Ng Wei Siang, Hugo Palmans, Yolanda Prezado, Judith Reindl, Francesco Romano, Loris Roncali, Aikaterini Rousseti, Thomas E. Schmid, Ileana Silvestre Patallo, Frank Stephan, Anna Subiel, Edward Taylor, Annalisa Walpen, Samuel Flynn

**Affiliations:** ^1^ Radiotherapy and Radiation Dosimetry Group National Physical Laboratory Teddington UK; ^2^ Center for Proton Therapy Paul Scherrer Institute (PSI) Villigen Switzerland; ^3^ Christie Medical Physics and Engineering The Christie NHS Foundation Trust, Manchester Academic Health Science Centre (MAHSC) Manchester UK; ^4^ Division of Cancer Sciences The University of Manchester Manchester UK; ^5^ Department of Medical Physics and Biomedical Engineering University College London London UK; ^6^ Danish Centre for Particle Therapy Aarhus University Hospital Aarhus Denmark; ^7^ School of Physics and Astronomy University of Birmingham Birmingham UK; ^8^ Datzmann Interact & Innovate München Germany; ^9^ Department of Physics Università di Torino Torino Italy; ^10^ University‐Hospital of Pisa Pisa Italy; ^11^ Department of Physics and Astronomy University of Manchester Manchester UK; ^12^ Department of Physics and Astronomy University College London London UK; ^13^ Department of Radiation Oncology Institut Curie, PSL Research University – Paris and Orsay Protontherapy Center Paris France; ^14^ Albert Einstein Center for Fundamental Physics (AEC) Laboratory for High Energy Physics (LHEP) University of Bern Bern Switzerland; ^15^ Deutsches Elektronen‐Synchrotron, Zeuthen site Zeuthen Germany; ^16^ National Institute for Nuclear Physics (INFN), Catania Division Catania Italy; ^17^ Institute of Applied Physics and Measurement Technologies Universität der Bundeswehr München Neubiberg Germany; ^18^ Department of Radiotherapy, Erasmus MC Cancer Institute Erasmus University Medical Center Rotterdam The Netherlands; ^19^ Department of Medical Physics & Informatics Holland Proton Therapy Center Delft The Netherlands; ^20^ Medical Physics, MedAustron Ion Therapy Center Wiener Neustadt Austria; ^21^ New Approaches in Radiotherapy Lab, Center for Research in Molecular Medicine and Chronic Diseases (CIMUS), Instituto de Investigación Sanitaria de Santiago de Compostela (IDIS) University of Santiago de Compostela A Coruña Spain; ^22^ Oportunius Program, Galician Agency of Innovation (GAIN) Xunta de Galicia A Coruña Spain; ^23^ University of Oslo Oslo Norway; ^24^ Department of Radiooncology TUM University Hospital Munich Germany; ^25^ Helmholtz Zentrum Munich Institute of Radiation Medicine Neuherberg Germany; ^26^ Department of Oncology University of Oxford Oxford UK; ^27^ Division of Medical Radiation Physics and Department of Radiation Oncology Inselspital, Bern University Hospital, and University of Bern Bern Switzerland

**Keywords:** minibeam therapy, particle minibeam therapy, PMBT workshop

## Abstract

Particle minibeam therapy (PMBT) has emerged as an advanced modality within spatially fractionated radiation therapy, using the physical properties of protons, ions, and electrons to achieve superior normal tissue sparing whilst preserving spatial dose distribution at depths that are unachievable in conventional kV or MV X‐rays. As with any novel radiotherapy technique, successful translation into routine clinical practice requires robust evidence of efficacy, safety, and technical feasibility. This review presents a coordinated roadmap for the development and future implementation of PMBT, identifying the key challenges that must be addressed to enable safe and effective clinical implementation. This review highlights the interconnected nature of PMBT research and proposes the priority areas for future investigation, and the steps necessary to guide PMBT from experimental concept, towards clinical implementation. As most of the steps are relevant to all particle types, PMBT will be referred to in general, where specific particle types will be discussed where appropriate.

## 1 INTRODUCTION

Particle minibeam therapy (PMBT) has gained much attention in recent years. As a form of spatially fractionated radiotherapy (SFRT), this modality is comprised of several sub‐millimeter particle beams delivering a non‐uniform dose at a shallow depth. This creates regions of high dose (referred to as “peak” regions), adjacent to regions of low dose (referred to as “valley” regions). Due to scattering, this can result in a homogeneous dose in the target region, however, the target dose may also be heterogeneous. For the purposes of this review, minibeam radiation therapy (MBRT), refers specifically to X‐ray minibeam radiation therapy and is itself a form of SFRT. PMBT represents the particle‐based subset of minibeam radiotherapy. A related technique, microbeam radiation therapy (MRT), uses much smaller beam widths (typically tens of micrometers) and is primarily implemented at synchrotron facilities. For completeness, the acronym pMBRT (proton minibeam radiation therapy), is noted as a specific subset of PMBT; it will only be referenced again where distinctions specific to this subset are being considered.

Spatially fractionated X‐ray GRID therapy, using millimeter‐sized beams, was first used over a century ago.^1^, ^2^, ^3^ It has been shown that its therapeutic efficacy improves as the beam size decreases.^4^ However, the smallest and most effective beamlets can only be generated at synchrotrons, with very limited patient accessibility and significantly high capital costs. More recent adaptations of SFRT techniques of GRID and lattice radiation therapy (LRT), use centimeter‐wide beams and have shown favorable tolerance profiles and good tumor control in early published results.^5^ These techniques were applied mostly in combination with standard radiation therapy (RT) for both curative and palliative treatments. The combination with particle beams is a developing technology that is regarded as highly promising for improving patient outcomes,^6^ benefiting from the advantages and accessibility of proton, ion, and electron beam therapy. Reproducible data showed that PMBT is associated with reduced acute and long‐term toxicity while anti‐tumoral effects were equal or superior to standard proton therapy in healthy and tumoral brain models.^7^, ^8^, ^9^, ^10^, ^11^, ^12^ Taken together, the preclinical data (Section 2) highlights the potential of PMBT to widen the therapeutic window in human cancer care. Moreover, PMBT seems to trigger specific biological mechanisms including the induction of an anti‐tumoral immune response.^13^ ^,^ ^14^ To date, there is no internationally agreed standard for measurement and reporting of radiation dose (Section 3). In addition, no patient PMBT treatments have been reported (Section 4), however, the potential is supported by the accumulation of positive clinical data from treatments with other spatially fractionated radiation therapy techniques.^15^

PMBT holds promise for tumors considered radioresistant such as high‐grade glioma and large brain metastasis,^16^ but also for conditions with narrow therapeutic windows and high probability of RT toxicity, such as pediatric tumors^17^ or reirradiation.^18^, ^19^ Additionally, combined treatments of immunotherapy and PMBT could take advantage of the immunomodulatory impact of the technique to improve both local and distant outcomes in tumors with high‐risk of metastatic spread, such as triple negative breast cancer.^20^, ^21^, ^22^

Preclinical research provides evidence of some underlying mechanisms that may lead to favorable outcomes for patients, but in‐human clinical trials remain the gold standard for clinical adoption. Commencing such trials in humans require careful consideration to ensure both the safety of enrolled patients and the production of meaningful data.

To accelerate the transition of PMBT from benchtop to bedside, the first international PMBT workshop was held in Paris, France, in 2023. The event aimed to promote knowledge exchange, stimulate discussion, and to collectively make steps towards clinical implementation. At the third international PMBT workshop, held at the National Physical Laboratory (NPL), UK, in March 2025,^23^ the community came together to develop this white paper, the primary objective of which is to provide a roadmap that identifies and prioritizes the key scientific, technical, and clinical challenges that need to be addressed to enable the safe and effective clinical adoption of PMBT. Perspectives from radiobiology, dosimetry, engineering, and clinical practice are presented in dedicated sections, reflecting the distinct constraints and opportunities within each field. While the focus is on PMBT specific requirements, topics that are more broadly valid for SFRT are described in that wider context.

## 2 RADIOBIOLOGY PERSPECTIVE

To truly exploit the benefits of PMBT, an understanding of the radiobiological implications of this modality is needed, spanning the underlying mechanisms and toxicity effects. Data from these studies can then be used to optimize the treatment and aid in clinical translation.

### 2.1 Toxicity in healthy tissues

The remarkable capacity of SFRT to spare normal tissues has been consistently demonstrated across various animal models, including mice, rats, and rabbits, as well as in multiple organs, such as the brain, bone, lung, and spinal cord.^15^ This tissue‐sparing effect is closely linked to the well‐established dose‐volume relationship, whereby a decrease in beam width leads to increased normal tissue tolerance.^24^

In PMBT, where broader beams (0.5‐1.0 mm) are employed, peak doses up to 100 Gy have been reported as well tolerated. However, toxic effects become apparent at peak doses of 150 Gy or higher, depending on beam width.^25^ Importantly, robust normal tissue sparing has been observed across several spatially fractionated particle modalities, including pMBRT,^10^ carbon minibeam radiation therapy (C‐MBRT),^26^, ^27^, ^28^ and neon ion minibeam therapy,^29^ in addition to previous observations with photon‐based techniques.^30^

The “cell migration and repopulation” hypothesis, supported by time lapse imaging studies and histopathological studies, proposes that non‐irradiated or minimally irradiated tissue regions preserve viable clonogenic cells capable of migrating into damaged areas and promoting tissue regeneration.^31^ According to this model, the preserved microdomains act as reservoirs of surviving cells that contribute to the repopulation of irradiated regions, thereby limiting necrosis and preserving tissue function. Additional mechanisms, including vascular preservation and maintenance of local tissue homeostasis, may also contribute to the enhanced normal tissue tolerance observed after spatially fractionated irradiation.

### 2.2 Mechanisms of PMBT

The biological effects in PMBT go beyond ‘simple’ cell death, where the effects appear more at the tissue/organ level, and systemic level. Proposed mechanisms of action for either MBRT or PMBT include differential vascular effects,^32^, ^33^, ^34^, ^35^ cell signaling effects,^36^, ^37^ inflammation, and immunomodulatory effects^11^, ^13^, ^38^, ^39^, ^40^ including abscopal effects at the tumor and healthy tissue level. Additional mechanisms include stem cell migration,^41^ generation and diffusion of free radicals, which may contribute to biological effects in valley regions in tumors.^42^, ^43^ In the following subsections, several proposed mechanisms and the existing experimental evidence and knowledge gaps will be briefly described.

#### 2.2.1 Immunomodulation

There is increasing evidence of the determinant role of the immune system in the anti‐tumoral response of SFRT.^44^ Recent studies^13^, ^14^, ^40^ have demonstrated the crucial role of T cells in the anti‐tumor response in SFRT by showing that the absence of efficient mature CD8^+^ T cells prevented the response to treatments, unlike immunocompetent animals. This was not observed in conventionally irradiated animals, irradiated at the same mean dose. An increased tumor infiltration by CD8^+^ T cells and B cells was observed following treatment. Enhanced infiltration of CD3^+^ T cells, without evaluation of the specific T‐cell subset, as well as CD4^+^ lymphocytes and natural killer cells, was also reported in primary Lewis mouse lung carcinoma tumors irradiated with LRT.^45^ Moreover, PMBT may be effectively combined with immunotherapy, where several promising results have been observed in preclinical studies, using different tumor models.^40^, ^46^

#### 2.2.2 Vascular impact

PMBT selectively targets tumor vasculature while sparing normal blood vessels, due to fundamental differences in vascular architecture, density, and biological resilience.^47^, ^48^ Normal tissue blood vessels are relatively sparse, spatially organized, and supported by stable structures including smooth muscle cells and pericytes.^49^, ^50^ As a result, most capillaries and small vessels lie between the minibeam paths, avoiding direct irradiation. Even vessels within high‐dose regions benefit from adjacent undamaged endothelium, which rapidly repopulates irradiated segments. Furthermore, normal vasculature is well oxygenated and equipped with efficient DNA damage repair mechanisms, enhancing its resistance to radiation‐induced injury.^51^

In contrast, tumor vasculature is dense, tortuous, leaky, immature, and structurally unstable, often lacking sufficient pericyte coverage and exhibiting poor perfusion. This disorganized and compact microvasculature increases the likelihood that many vessels intersect minibeam paths and receive lethal localized doses. Endothelial cells in these vessels sustain extensive radiation damage leading to apoptosis, vascular collapse, and hemorrhage.^52^, ^53^ Tumors also exhibit a reduced capacity for vascular repair due to an unfavorable microenvironment and limited access to normal tissue repair mechanisms.

Moreover, SFRT induces differential molecular responses between tumor and normal tissues. Tumors show upregulation of pro‐inflammatory cytokines such as TNF‐α and IL‐6, along with endothelial adhesion molecules like ICAM‐1, which amplify the vascular response and increase radiosensitivity. Prezado et al. demonstrated that SFRT enhances tumor vascular permeability by activating inflammatory signaling pathways, facilitating therapeutic agent extravasation and exacerbating vascular disruption.^6^

The predominance of immature vessels in tumors correlates to increased capillary rarefaction, enlarged inter‐vessel distances, and reduced vessel density and perfusion after SFRT. These pathological changes are notably dissimilar to the relatively preserved perfusion, blood volume, and vascular density in normal tissues.

Therefore, PMBT may selectively disrupt immature, disorganized tumor vasculature while sparing mature, stable normal vessels, leading to enhanced tumor vascular damage and differential molecular responses that aid tumor control. Nonetheless, critical gaps remain in fully understanding the molecular mechanisms driving these vascular effects, especially the roles of inflammatory signaling and permeability to molecules of intermediate size. Further research is essential to characterize how vascular maturation influences radiosensitivity, clarify vascular repair kinetics and explore combination therapies, leveraging increased vascular permeability to improve clinical outcomes.

#### 2.2.3 Vascular and immune dynamics in response to ionizing radiation

Irradiation of the tumor vasculature can induce vasculogenesis, increased vascular permeability, reduced pericyte coverage^54^ and expression of adhesion molecules like VCAM‐1 and ICAM‐1 on the endothelial cells surface.^55^ The latter facilitates immune cell adhesion and subsequent diapedesis into the tumor.^56^ Access of circulating T cells can be enhanced after RT because of changes in pericytes‐associated markers like NG2 and aSMA.^57^

Understanding these complex interactions between ionizing radiation, endothelial cells, and immune infiltration and how they can be modulated by altering dose delivery methods (conventional versus MBRT, homogenous dose distributions versus heterogeneous doses) is vital for optimizing cancer therapy. Interpreting the complex interplay within the tumor microenvironment (TME) including the endothelial response, lymphocyte, and myeloid cell infiltration, and how they impact spatial dose modulation is key for further developments of MBRT.

#### 2.2.4 Bystander effects

Bystander (‘cohort’) effects following SFRT irradiation have been demonstrated in both in vitro and in vivo studies, although mechanistic investigations have been performed predominantly in vitro.^36^, ^37^ In vivo evidence includes increased TNFα levels observed after GRID irradiation in patients^58^ and in murine Lewis lung carcinoma models treated with LRT.^45^ In addition, increased expression of genes involved in DNA repair, cell cycle arrest, heat shock response, and apoptosis have been reported in valley‐region cells following GRID irradiation.^36^, ^37^ Bystander effects have also been observed in MRT, both in vivo and in vitro.^59^, ^60^ Some beneficial bystander‐mediated responses have been hypothesized to contribute to normal tissue sparing.^61^ Supporting indirect evidence includes increased turnover of heat shock protein 71 (HSP71) in the contralateral non‐irradiated brain hemisphere of irradiated rats,^59^ a response that has been associated with protection against neurological damage.

#### 2.2.5 Free radicals

Free radicals are known to induce direct and indirect cellular damage, with DNA as a prime target.^62^ Understanding how PMBT generates free radicals and its role in cell death can significantly enhance the clinical potential of PMBT. Studies using Monte Carlo (MC) and TOPAS‐nBio simulations have shown that radical types and quantities differ between peak and valley dose regions.^42^, ^43^, ^63^, ^64^ Dal Bello et al. identified hydrogen peroxide as a radiolysis product that stably diffused to fully cover the dose valley with a strong oxidizing property, serving as a suitable candidate among reactive oxygen species (ROS) involved in PMBT efficacy.^42^ Masilela et al. demonstrated that radiation quality significantly influences ROS levels in MBRT, with heavy ions producing more radicals in valleys as linear energy transfer (LET) increases.^43^ Consequently, ROS led to cellular damage through both the targeted and non‐targeted effects mediated through several cellular processes such as cell signaling, bystander effects, DNA damage, lipid damage, eventually inducing cell death. However, evidence suggests that the dose heterogeneity inherent to PMBT, reduces the oxygen dependency of the tumors, thus indicating the potential efficacy of PMBT in the treatment of hypoxic tumors.^65^

### 2.3 Multimodality treatments

The unique physical and biological effects of SFRT make it a promising modality for combination therapies, with the potential to improve therapeutic outcomes.^8^ Pro‐inflammatory and immunogenic microenvironment triggered by PMBT can synergize with immunotherapies, such as immune checkpoint inhibitors, to potentiate antitumor immune responses.^66^ Combining SFRT with targeted molecular therapies may further exploit tumor‐specific vulnerabilities, such as defective DNA repair pathways or aberrant signaling networks, thereby enhancing radiosensitivity and tumor control.

MBRT and PMBT could be integrated with other treatment modalities to broaden its clinical impact.^8^ Recent preclinical studies demonstrate that combining MBRT with immunotherapy,^66^ chemotherapy, advanced particle therapy techniques and high dose rate (“FLASH”) delivery can increase tumor control or normal tissue sparing,^67^ beyond what is achieved with MBRT alone.

#### 2.3.1 MBRT plus immune checkpoint blockade

A study by Rivera et al. tested kV X ray MBRT combined with anti PD L1 checkpoint inhibition in a dual tumor murine mammary carcinoma model. A minibeam peak/valley dose of 50 Gy/3.7 Gy produced a stronger abscopal (distant tumor) response than a peak/valley dose of 100 Gy/7.4 Gy and was significantly more effective than uniform radiation therapy plus anti PD L1 (*p* = 0.04948). Flow cytometry profiles showed that MBRT preserved resident CD3^+^ and CD8^+^ T cell populations and, when paired with PD L1 blockade, increased systemic CD8^+^ T cells. These results suggest that the low dose valley regions (<5 Gy) permit immune cells to survive and migrate, turning the irradiated tumor into an in situ vaccine that synergizes with checkpoint blockade.^22^

#### 2.3.2 MBRT mediated microenvironment reprogramming

MBRT was observed to induce spatially fractionated dose distributions with high peak‐to‐valley dose ratios (PVDR) in triple‐negative breast cancer using a 3D‐printed collimator. MBRT significantly reduced tumor proliferation and hypoxia compared with conventional radiotherapy at identical valley doses. On a mechanistic level, MBRT attenuated the hypoxia‐inducible factor 1‐alpha (HIF‐1α)/vascular endothelial growth factor receptor (VEGFR) signaling, resulting in inhibited angiogenesis, enhanced pericyte coverage, and enhanced vascular architecture in line with vascular normalization. The vascular alterations reduced tumor hypoxia and indicated that MBRT has the potential to potentiate angiogenesis and hypoxia‐relevant therapies.^68^

In agreement with these results, MBRT also improved the delivery of chemotherapy. Fazzari et al. discovered that spatially fractionated irradiation facilitated intratumoral drug delivery and penetration compared with standard irradiation.^35^ This was also observed in the context of enhanced vascular functionality and lower interstitial barriers allowing for more chemotherapeutic agents to build up in the tumor microenvironment.^69^ This supports the hypothesis that vascular remodeling induced by MBRT modulates tumor oxygenation while also improving systemic therapy delivery and efficacy.^70^

### 2.4 Open questions

To support the clinical translation of PMBT, there are several unanswered questions that will aid in the optimization of this modality. Some of these include investigating the differential effects between PMBT and MBRT or the effects on healthy tissues to determine the normal tissue sparing mechanisms. Due to the complexity of the various factors associated with PMBT (particle type, dose rate, heterogeneity in dose delivery etc.), clear descriptions of the experimental conditions are crucial for comparisons to be effectively made between different studies. The ideal scenario would conduct a parametric study to determine the biological impact of each individual parameter, replicated and validated at independent institutes. Another crucial aspect is that many studies have been conducted in research facilities, where there is a growing need for more preclinical studies to be performed under clinically applicable conditions. Findings from these could then be utilized to optimize the conditions and demonstrate the true benefits of PMBT.

## 3 DOSIMETRY PERSPECTIVE

The method of measuring and reporting radiation dose in PMBT is yet to be resolved, with discrepancies in the approach varying between centers. Standardization of the dosimetric methodology is required before routine clinical implementation.

### 3.1 Parameters of interest

Parameters relevant to PMBT are categorized as either geometric, or dosimetric.^71^ The extent to which these geometric and dosimetric parameters influence the biological response remains not fully understood.^71^ The complex nature of PMBT, characterized by pronounced dose gradients, introduces challenges for dosimetry.^72^, ^73^, ^74^ Tissue heterogeneities further compound these dosimetric challenges.^75^ In particular, the influence of heterogeneities such as bone and lung on peak broadening, valley‑dose elevation, and the preservation of spatial modulation remains an area requiring further investigation. While Monte Carlo methods remain the most robust approach for modelling particle transport in PMBT, their application to ultra small fields and heterogeneous media require additional validation to confirm the reliability of estimated peak and valley doses under these conditions.^76^ At present, there is no consensus regarding the minimum set of parameters that need to be reported for a complete clinical description.

#### 3.1.1 Dosimetric parameters

Frequently reported dosimetric parameters include:

- Peak dose (PD), valley dose (VD), and PVDR. Additional quantities such as average dose (AV‐D), integrated dose (ID) and flatness have also been reported with multiple definitions. Definitions of PD encompass: the maximum dose within the high‐dose regions, peak prominence (representing the dose difference between a peak and its lowest contour line),^75^ and the average along a line profile positioned on each peak.^77^ VDs are analogously defined through various approaches.

The PVDR is likewise subject to several definitions. Examples include the average peak dose divided by the average valley dose across all peaks and valleys within a dose profile^78^, ^79^ or for each individual peak,^75^ the ratio between the central peak only and its adjacent valleys,^80^ or a single representative peak and valley dose derived from the average of dose profiles from multiple measurements at the same depth.^77^ AV‐D might be interpreted as the volume‐average dose^74^, ^80^ or as the average between the first and last peaks across a surface profile.^13^ Given the highly heterogeneous dose distributions inherent to PMBT, a quantity reflecting the variation among peak doses holds considerable interest. For instance, Kim et al. defined flatness as ($\frac{Dp_{max}-Dp_{min}}{Dp_{max}+Dp_{min}}\times 100)$, where $Dp_{max}$ and $Dp_{min}$ denote the maximum and minimum peak doses, respectively.^79^ In highly spatially fractionated beams, the VD may approach zero gray, which causes the PVDR to increase without bound. To avoid this numerical instability, an alternative quantity known as the valley‐to‐peak dose ratio (VPDR) has been proposed.^81^ VPDR takes values between zero and one, making it a more stable and interpretable parameter in situations where the valley dose is extremely low. An infrequently reported dosimetric metric is Bragg‐peak‐to‐entrance dose ratio,^82^ which is useful when comparing PMBT surface‑dose‑related toxicities or assessing the relative sparing of entrance tissues. As it is currently unclear which of these parameters are essential, it is recommended to report all of those listed (PD, VD, AV‐D, ID and flatness), providing for each a clear description of how it was determined.

ii. Dose rate: different definitions of dose rate should be distinguished; the explicit bunch‐averaged dose rate (dose rate of the individual particle bunches), the pulse‐averaged dose rate (dose rate within the RF pulse), and the average dose rate (total dose divided by total treatment time). Until further clarity is available it is recommended to report all these dose rates, providing for each a clear description of how it was determined.^83^ Moreover, thinking about a possible synergistic effect with the FLASH effect, which is triggered by both dose per pulse and dose rate thresholds,^84^, ^85^ it is necessary to define: the peak zone and average dose rate, dose‐per‐pulse and instantaneous dose‐per‐pulse within this zone (particularly for minibeams obtained by dose painting with pencil beam scans).
iii. Homogeneous versus heterogeneous distributions at the target: depending on the configuration used for PMBT, the target (Planning Target Volume (PTV)) is irradiated with homogeneous or heterogenous dose distributions, which impacts the way the mini beam effect is exploited. The authors recommended that the level of homogeneity in the target region is described by a homogeneity index or by the PVDR in at least three depths within the target region (proximal, central, distal).

#### 3.1.2 Geometric parameters

Examples of geometric parameters commonly used to evaluate preclinical MBRT and PMBT plans that use fixed collimators are^86^, ^87^: (i) collimator gap width (open beam segment width), (ii) center‐to‐center spacing between beam segments, (iii) collimator‐surface‐distance (alternatively described as the phantom‐to‐collimator distance^88^).

These parameters describe the physical geometry imposed by the collimator and determine the initial beam layout before interaction with tissue or scattering processes. In addition, radiation‑field‑derived spatial parameters are often reported because they characterize the pattern formed in the irradiated volume. These include, for example: (iv) valley width, (v) the percentage of the volume encompassed by peak regions, and (vi) the full width at half maximum (FWHM) of the minibeam, potentially at different depths, which reflects the effective beam broadening resulting from scattering, energy loss, or delivery technique.

Similar geometric parameters are commonly recorded for clinical GRID SFRT radiotherapy studies (using commercial or bespoke designed collimators). In the case of LRT, (i) number of vertices, (ii) median center‐to‐center distance between nearest neighbors’ vertices, (iii) Gross tumor volume (GTV) volume and (iv) vertices‐to‐GTV volume ratio are among the most cited.^89^

For plans involving multiple PMBT fields from differing or non‑coplanar beam directions, the reporting should include the beam‑incidence angles and the three‑dimensional spatial coordinates defining each field's placement (e.g., entry point or isocenter position).^90^

Finally, although not currently frequently reported parameters, microdosimetric quantities may provide beneficial information for comparing configurations and demonstrating biological effectiveness, as shown in several Monte Carlo studies.^64^, ^91^, ^92^ The energy distribution and other microdosimetric quantities vary across and along the PMBT patterns, which could correlate with the radiobiological outcomes. Specifically, microdosimetric spectra and related average quantities could be measured to account for the different beam quality at the peaks and the valleys for example to account for changes in energy spectrum due to secondary particles produced at the edges of minibeam collimators.

### 3.2 Reference dosimetry

Following established codes of practice such as the IPEM Code of Practice for Proton Therapy,^93^ TRS‐398^94^ or AAPM TG‐51,^95^ reference dosimetry aims to measure the absorbed dose under reference conditions that reflect the dose delivered to the target in a clinical environment. For conventional radiotherapy, the reference field is a large, uniform, 10 × 10 cm^2^ beam, with the measurement point situated within a region of charged‐particle equilibrium and minimal dose gradient. These conditions are designed to ensure reproducibility and consistency across institutions.

In PMBT, however, treatment fields are inherently non‐uniform and often consist of spatially modulated, high‐gradient patterns.^88^, ^96^ If the dose within the target region is reasonably uniform, for example when a spread‑out Bragg peak is combined with a PMBT field that relies on multiple Coulomb scattering to eliminate spatial fractionation before the beam reaches the target,^79^ then reference dosimetry can be performed directly in that target region or in the closest available region where the dose remains uniform and free of strong gradients.

If, however, the dose is strongly non‐uniform in the target region, an approach deviating from that of conventional radiotherapy is required as previous assumptions essential for conventional reference dosimetry (field uniformity, lateral charged‑particle equilibrium, and negligible volume‑averaging distortion) no longer hold. This may need new approaches in compiling preclinical and clinical data, such as that proposed by Bassler et al.^97^ in their work on calibrating monitor units in terms of biological outcome or Elbanna et al. in their work on personalized radiation dose.^98^

As reference fields for absolute dosimetry, a range of options may be considered. This roadmap does not assert which, if any, of these approaches will ultimately prove most suitable for long‐term PMBT clinical adoption; rather they are presented as candidate methods for consideration. Several examples are listed here, although this is not intended to be an exhaustive list:

- an open field created by removing the mechanical collimator or by disabling the focusing mechanism that realizes the SFRT field. This produces the largest uniform field that the beam delivery system can deliver and allows reference dosimetry to follow TRS‐398^94^ with only minor adaptations if the system cannot generate a full 10 × 10 cm^2^ field. While this is likely the most accurate way of performing reference dosimetry, as it satisfies the assumptions of field uniformity and charged‐particle equilibrium, the disadvantage is that the correlation between the reference field and the clinically delivered fields is low. Therefore, relative dosimetry of the SFRT fields with respect to the reference dosimetry will bear a large uncertainty.
- a point dose measurement in the non‐uniform region of a representative SFRT reference field against which relative dosimetry can be conducted. For GRID or LRT this is a viable option, and the dosimetry problems can be reduced to those of small‐field dosimetry (e.g., IAEA TRS‐483^99^ for photons). Given the very steep dose maxima present in many other PMBT applications this may not be an option.
- average dose over a well‐defined area of a representative PMBT field perpendicular to the beam direction. This option has been studied by Cotterill et al., showing that this is possible, but revealing that lateral positioning uncertainty can be a dominating contribution to the uncertainty.^100^ A considerable difficulty is the geometrical matching of standards (e.g., calorimeter) and reference dosimeters (e.g., ionization chambers).
- average dose over a well‐defined volume of a representative PMBT field. This option could reduce some of the uncertainties of the area approach but still be prone to the difficulty of geometrical matching.
- laterally integrated dose of a single slit, multiple slits or the entire field of a representative PMBT field using a detector which is sufficiently large to capture the entire lateral profile. This option reduces positioning uncertainty and enhances reproducibility but would only be possible in fields that are not too large.
- a dynamically acquired integrated dose of a single slit, multiple slits or the entire field of a representative PMBT field by scanning a detector through the field. The method is similar to the approach previously demonstrated by Prezado et al. for microbeam radiotherapy.^101^ It is reciprocally equivalent to the previous method similarly as the dose‐area‐product for a single pencil beam in scanned particle beams is derived from a point dose measurement in a pencil beam scanned field.

### 3.3 Relative dosimetry

For PMBT beams, it is no different from conventional radiotherapy that ideally a full 3D dose distribution is known to support prescriptive radiation treatment. For conformal radiotherapy, this is achieved through the use of output factors, lateral profiles and percentage depth doses for a representative set of fields in order to create a beam model, which can be incorporated into a treatment planning software (TPS). With increasing complexity of radiotherapy delivery, fluence‐based or particle‐number‐based dose calculation methods or approaches based on full Monte Carlo simulations of the beamline are increasingly used instead of model‐based dose calculations.^102^ Consequently, the approach to relative dosimetry has shifted to measure the parameters necessary to commission these dose calculation systems, such as small field profiles and output factors, penumbrae, and verifying the dose calculation system for more complex dose distributions as part of commissioning.^103^, ^104^

As PMBT necessarily relies on Monte Carlo dose calculation or other sophisticated dose calculation engines, a similar approach is appropriate. Given the vast variety of possible SFRT applications, it is difficult to provide a generic recommendation, but a non‐exhaustive list is presented in this roadmap of how it could be implemented in PMBT, with the community to investigate, assess, and evaluate which, if any, are the most suitable.

There are two main experimental conditions:

- Open field conditions: As described in Section 3.2, an open field created by delivering the beam without any collimation or magnetic focusing can serve as the reference field for absolute dosimetry when a uniform region is unavailable within the PMBT configuration. In relative dosimetry, this same open field provides the baseline against which measurements in the PMBT field are normalized. Relative dosimetry then proceeds using detectors capable of resolving the steep dose gradients characteristic of PMBT fields to acquire appropriate measurements. The resulting measurements are subsequently referenced to the open‑field calibration by applying the appropriate tabulated or Monte Carlo derived correction factors. Although this approach resembles the methodology described in IAEA TRS‐483^99^ for dosimetry of “small beams”, it must be acknowledged that PMBT minibeams are far smaller than the centimeter‐scale fields addressed in that code of practice and thus require careful interpretation. The detector used for reference dosimetry need not be the same as that used for relative dosimetry.

The relative dose in a PMBT field ($\Omega_{Q_{clin},Q_{ref}}^{f_{clin},f_{ref}}$) following TRS‐483 formalism is therefore given by:

$$\Omega_{Q_{clin},Q_{ref}}^{f_{clin},f_{ref}}=\frac{M_{Q_{clin}}^{f_{clin}}}{M_{Q_{ref}}^{f_{ref}}}\;k_{Q_{clin},Q_{ref}}^{f_{clin},f_{ref}}$$

where $M_{Q_{clin}}^{f_{clin}}$ is the detector reading in the PMBT field, $M_{Q_{ref}}^{f_{ref}}$ is the detector reading in the open‐field reference (Section 3.2), and $k_{Q_{clin},Q_{ref}}^{f_{clin},f_{ref}}$ is the output‐correction factor.

ii. Measurements for validation of Monte Carlo tools: To validate the Monte Carlo simulations required for PMBT dose calculation, measurements should be performed at well‐defined depth using detectors with sufficiently high spatial resolution. For low energy electrons, as an example, this depth may correspond to the surface or the depth of maximum dose. The simulations should be performed in at least two configurations. The first configuration reproduces the open‐field reference condition defined in Section 3.2. This provides a normalization factor for the computed dose distributions in terms of absolute dose, starting from a specific and arbitrary number of generated events. The second configuration reproduces the measurements of the PMBT experiment. Continuing the example for low energy electrons, this should include the minibeam shaping components, whether mechanical collimator or magnetic scanning system, and be done at the same depth as the experimental measurement. The resulting dose distribution from this second simulation is then normalized to that obtained using the open‐field condition.

Additionally, measurements of the average dose across the minibeam pattern at the same depth will be required, as well as the percentage dose distribution. The related region of interest for these measurements must be properly established in terms of size and shape, according to the specific minibeam pattern used. These averaged quantities ensure that the measured features of the PMBT field can be accurately aligned with the corresponding Monte Carlo results.

### 3.4 How to measure the required quantities

The experience gained with conventional irradiation delivery devices and patient‐specific quality assurance (PSQA) can be translated to the specific requirements of PMBT. Measurements of depth dose curves and off‐axis dose distributions are well established, in addition to the use of radiochromic films and 2D arrays within phantoms.^105^ Local quality assurance (QA) programs for the different SFRT modalities have been covered by several groups.^106^, ^107^

New detector technologies are being developed to support QA in PMBT, addressing the challenges posed by steep dose gradients and high dose rates.^108^, ^109^, ^110^ In addition, bespoke phantoms (as test objects for dosimetric verification) must be developed for PMBT to accommodate these new types of arrays. The combination of these innovations is essential for enhancing PSQA, ensuring accurate treatment delivery, and reducing uncertainties in complex PMBT techniques.

Suitable detectors require the following characteristics for dosimetry in PMBT:

- high spatial resolution for minibeam dose distribution measurements (<100 µm)
- high sensitivity for the accurate measurement of the valley dose (low dose regions)
- wide dose range sensitivity to simultaneously measure the peak and the valley dose
- independence of the response from the energy spectrum and from the LET

Table 1 lists various detectors that have been either considered for use in PMBT or have been evaluated with SFRT and could be applicable to PMBT, showing how they match the requirements above and their respective advantages.

**TABLE 1:** Summary of detectors considered for use in PMBT.

Detector	Spatial resolution	Temporal resolution	Advantages	Disadvantages	Additional comments
Radiochromic Film^111^, ^112^	<25 µm depending on scanner resolution and film type	Not real time, 24‐h needed as processing time	2D imaging, high spatial resolution, considered energy independent over clinical energies	Single use. Scanning procedure and conditions are critical to the result. High LET causes quenching. Careful handling required to avoid scratches and defects	New methodology proposed with optical fiber probe for real‐time readout^113^
Ionization Chambers^29^, ^100^	Sizes: mm‐cm	Real time measurement	Commonly used in radiation therapy, established training. Suitable to evaluate broad beam conditions. Traceable	Low spatial resolution. Averages over the measuring volume. Positioning inaccuracies. No suitable protocols for minibeams	
Pixelated solid state Detectors Silicon/Diamond/Silicon carbide^109^, ^114^, ^115^, ^116^	<100 µm	Real time response	Minimal directional dependence <0.65%	Requires radiation quality correction factors. Sensitive to cumulative radiation damage. Energy dependency	
Scintillating fiber^117^	<100 µm (fiber)	Real time response	Water equivalent, inexpensive	One dimensional. Need to move to scan the beam. Energy dependency	
Strip detectors^118^, ^119^	<100 µm	Real time response	Fast, real time High resolution images	Sensitive to cumulative radiation damage. Sensitive to position misalignment. Energy dependency	
Scintillator coupled with a camera^120^	<1 mm	Real time response	Can be dose rate independent^121^	Energy dependency	
Gel^122^	µm ‐mm (depending on gel type and imaging technique used)	Passive, non‐real time	3D dose imaging capability. Dose rate independence (for NIPAM)	Passive detector that requires post processing. Sensitivity to oxygen concentration. Requires careful handling. Potential toxicity during preparation and disposal. Quenching effects	NIPAM polymer gel dosimeter used for 3D verification of LRT
Ionoacoustic^123^, ^124^	< 1 mm	Real‐time measurement	Non‐invasive /Passive. Real‐time. Potential for 3D dose mapping	Very weak acoustic signals would need to be detected. Short beam pulses are required to generate ultrasound signals. Currently unexplored for minibeam detection	
Cherenkov/ Optical^125^	N/A	Real‐time image	Can be used to optimize beam focusing	Used for qualitative visualization only Viewpoint can cause image distortions meaning quantitative information cannot be extracted	
Calorimeters^100^	mm‐cm	Real‐time measurement	Could serve as dosimetric standard. High accuracy (for very sophisticated and complex designs)	Instruments typically owned by standards labs. Operation and analysis requires specialist knowledge. Poor spatial resolution. Only measures average dose over a defined area. Positioning inaccuracies significantly increase uncertainties	Primary standard graphite calorimeter used in cited work

Due to their spatial complexity, special care is needed at the time of performing reference and QA measurements in SFRT. Uncertainties related to the positioning and orientation of the detectors and reproducibility of the collimated minibeams might have a large influence in the determination of absorbed dose and dose distributions.^100^

## 4 ENGINEERING PERSPECTIVE

In conventional particle therapy, dose delivery is achieved either via passive scattering or pencil beam scanning using focused beams. In passive systems, the beam is shaped and spread by a series of scattering foils, range shifters, rotation wheels and collimators.^126^ In pencil beam scanning, scanning magnets are used to raster the focused cm‐sized pencil beam across the target volume. The fundamental principles of both techniques can be adapted to PMBT, where the key requirement is a sub‐millimeter beam size and a spatially modulated fluence distribution.^96^

### 4.1 Magnetically focused beams

PMBT, using magnetically focused beams, involves the use of specialized magnetic optics—most notably quadrupole magnets—to demagnify the beam size. By carefully tuning the beam optics, a broad initial beam can be transformed into a tightly focused pencil or planar beam with sub‐millimeter widths, which can be scanned over the desired field at the patient. This approach offers key advantages that position it as the ideal solution for PMBT.^127^, ^128^ In particular, the beam is not physically obstructed, and the beam current is preserved, which is crucial for integrating emerging ultra‐high dose rate delivery techniques such as FLASH RT. Furthermore, magnetic focusing avoids the introduction of additional scattering sources, eliminating secondary radiation production due to the collimator, and the consequential increase in valley dose that can compromise tissue sparing. The magnetic system's tunability also allows rapid switching between different minibeam configurations without mechanical adjustments, supporting flexible treatment plans and adaptive therapy. Preclinical studies using focused minibeams have already been conducted at the ion microprobe SNAKE (Superconducting Nanoprobe for Applied Nuclear (Kern‐)physics Experiments) in Garching (Germany), showing reduction of side effects compared to homogeneous irradiation.^11^, ^129^

However, these advantages come with substantial technical and economic challenges. Clinical facilities today lack the dedicated beamline optics necessary to achieve the required sub‐millimeter beam sizes at treatment depth. Constructing such systems requires the design and installation of high‐gradient magnetic lenses with precise control and stability, demands major modifications to existing beamlines or the development of new research‐focused beamlines.^130^ Additionally, the calibration, QA, and beam monitoring systems must be adapted or redeveloped to characterize and maintain these highly focused beams accurately. Due to these complexities, magnetic focusing for PMBT remains in the research and development stage, with no current clinical installations capable of delivering sub‐millimeter minibeams via focusing. The most advanced system, the MiniBEE (Minibeam BEamline for pre‐clinical Experiments), is currently being built at the Helmholtz‐Zentrum Berlin.^131^, ^132^ This system will focus a 70 MeV proton beam to ∼50 µm (σ) beamspot size. It will be the first system capable of systematically focusing high‐energy proton beams to such small sizes, serving as a platform for preclinical studies and engineering developments in beam delivery, dose‐monitoring and beam application modes. Systems like that are a crucial step towards clinical implementation. Furthermore, the timeline for clinical translation depends on sustained investment in beamline engineering, commissioning, and regulatory approval processes. It is within the next 10 to 15 years, contingent upon the successful construction of dedicated beamlines, thorough preclinical validation, and early‐phase clinical trials. Magnetic focusing is a highly flexible technology for particle minibeam production and fulfills all desired requirements in terms of application times and pattern, whilst avoiding secondary radiation. However, its technical complexity and challenging implementation make it a long‐term goal and make it unsuitable for the first implementation of PMBT into the clinic.

### 4.2 Mechanical collimation

**FIGURE 1:**
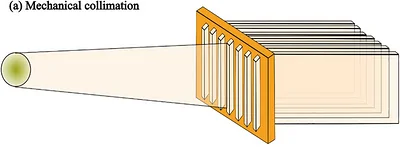
Planar minibeam generation through mechanical collimation. Green: Beam profile of a divergent beam at the start. Dark orange: Multi slit collimator. Light orange: Beam envelopes. Several planar minibeams are produced by either scanning of a pencil beam or using a large incident beam.

Mechanical collimation (Figure 1), as the second option, achieves beam size reduction by physically blocking parts of the beam using multi‐slit apertures or grids positioned near the patient or within the treatment nozzle. This technique is well‐established in MBRT^77^, ^133^, ^134^ and has been adapted for particle therapy,^135^ with promising preclinical results.^136^, ^137^

The transportable multi‐slit collimation system developed by Ahmed et al.^73^ is a prime example of such a device, designed as a modular multi‐slit collimator compatible with existing clinical proton therapy nozzles and used for preclinical experiments at the University Proton Therapy Dresden, Germany^138^ and the University of Washington Therapy Center, USA.^139^ Its precision‐manufactured slits generate arrays of proton minibeams with consistent widths (e.g. 250 µm) and spacing (e.g. 1 mm center‐to‐center (ctc)), producing high PVDR, crucial for sparing normal tissue.

Mechanical collimation offers the practical advantage of rapid integration into existing therapy rooms without requiring extensive beamline redesign.^140^ This compatibility makes it an attractive candidate for early clinical translation. However, several technical challenges accompany this approach. First, blocking part of the beam leads to significant current loss, reducing the achievable dose rate and precluding the immediate use of FLASH modalities. Second, particle interactions within the collimator material generate secondary particles and scattered particles, increasing the valley dose and complicating dose distributions. This secondary radiation requires careful dosimetric characterization and enhanced radiation protection measures. Finally, collimators are typically static and customized for particular beam configurations, limiting flexibility.^130^ Ongoing improvements to collimator design focus on optimizing slit geometry and materials to minimize scattering and secondary radiation.^137^

### 4.3 Integrating production techniques into clinical practice

Weighing the advantages and disadvantages of both production techniques, we conclude that for rapid clinical translation, collimation is the pathway to be followed, while not losing the focus on further development of magnetically focused beams, as in the long term, this option is highly flexible, versatile in terms of beam patterns and superior regarding dose delivery.

To initiate a clinical trial with collimated minibeams, several obstacles must be addressed. On the hardware side, integrating collimators into existing nozzles requires mechanical support structures, accurate alignment mechanisms, and beamline shielding evaluations. We suggest developing and using a support system that can easily fit into various gantry types, together with a standardized collimator in the first clinical trials. This has the advantage that dosimetry can be done in detail at a research gantry for the system in general and a QA protocol can be developed for use in all other centers, to allow fast integration and have the least impact on the clinical routine. For full characterization, facilities like the National Physical Laboratory (NPL) in the UK can use advanced dosimetry tools, like calorimeters, to obtain a comprehensive, traceable dosimetric characterization of the beam, which can serve as a beam model for treatment planning.

In terms of the software, TPS must be adapted to handle spatially modulated fields, including realistic beam modeling, MC simulations, and biological effect modeling. Currently, one of the most significant limitations is the lack of experimental reference data for benchmarking dose distributions, beam interactions, and tissue response. Without validated models, treatment planning output remains uncertain, limiting clinical confidence. Therefore, a concerted effort must be made to generate high‐quality dosimetric and biological data to inform and validate treatment planning.

### 4.4 Practical considerations for PMBT delivery

The time of delivery for a scanning treatment differs from having a dose delivery through a mask. This causes different dose rates and can impact the biological effect. If there are very short total radiation delivery times, with possible beneficial biological effects (e.g. FLASH effect), it may not be possible to stop irradiations within the treatment time. Therefore, the stability of radiation delivery must be high enough to trust the “single shot”.

Active scanning beams result in minimal beam loss, while passive collimation may discard more than half of the beam.^141^ This not only increases treatment time but also contributes to collimator activation and secondary neutron production.^142^, ^143^, ^144^ In passive PMBT, activation of collimators should be considered. Collimators are typically fabricated from high‐Z materials such as brass or tungsten, to achieve sufficient beam shaping.^88^ However, these materials become activated under particle irradiation.^145^ Tungsten is effective for beam collimation but exhibits longer decay times compared to brass.^146^ Among brass alloys, those composed purely of Zn and Cu are preferable, to minimize the production of long‐lived radionuclides.^146^ While the activation risk to patients is negligible compared to the dose from treatment, cumulative exposure to activated collimators represents a concern for clinical staff involved in repeated treatment setups throughout the day. Mitigation strategies include using internal self‐shielding designs, or alternating between multiple collimator sets to allow for decay between uses.

The ability to deliver a spatially fractionated dose to the target is limited by the physics of particle beams.^128^ As the beam travels through a medium it broadens for two reasons:

- Beam optics: The optics can be controlled by the design of the beam delivery system. E.g. The spot‐size and divergence of the beam may be controlled via beamline design, or the spot‐size may be sharpened by introducing collimation.
- Multiple Coulomb scattering: A particle beam inherently broadens with depth as it travels through the medium. This broadening cannot be controlled by the design of the delivery system.

Multiple Coulomb scattering makes it difficult to maintain high PVDRs for millimeter‐scale beams as the target depth is increased. A planning study by Charyyev et al. investigated the use of a brass collimator as a means of producing millimeter‐scale proton beams with a clinical pencil‐beam scanning system for delivery of PMBT.^147^ The resulting PVDR was dependent on depth and beamlet spacing. They concluded that 2 mm beamlets are feasible using current clinical technology.

Centimeter‐scale SFRT is less constrained by the beam broadening which results from multiple Coulomb Scattering, and its clinical implementation is more advanced than PMBT. The similarities in the two approaches, notwithstanding the differences in physical scale, mean that clinical data and outcomes from SFRT are relevant to PMBT and may be useful to guide its clinical development. There may also be future studies which leverage the similarities between the techniques, and any clinical benefits shown for SFRT may prompt further interest in PMBT.

## 5 CLINICAL PERSPECTIVE

Carefully designed clinical trials are required to demonstrate the efficacy of PMBT in patients. Considerations for trial design include palliative versus curative settings, disease type/location, tumor control/toxicity endpoints, treatment planning/delivery strategies, QA procedures, and accurate dosimetry.

### 5.1 Types of irradiations that can be prescribed (target coverage)

SFRT has been primarily applied in clinical settings for palliative treatment using techniques such as GRID and LRT.^15^, ^148^, ^149^, ^150^ In contrast, PMBT and MBRT have so far remained largely restricted to preclinical research.^15^ A recent milestone was achieved in 2024, when Grams et al. conducted the first clinical application of MBRT using patient‐specific 3D‐printed collimator holders in two patients, one with Merkel cell carcinoma and another with recurrent preauricular squamous cell carcinoma.^77^ Both patients experienced rapid pain relief and measurable tumor responses after only two treatment fractions, demonstrating the clinical feasibility of MBRT. Jacobson et al. also reported on the use of MBRT with an orthovoltage unit employed as a compassionate treatment in a patient with recurrent mucosal melanoma, showing a dramatic tumor response and minimal toxicity more than 6 months post‐treatment.^151^

Preclinical evidence for SFRT is expanding across a wide range of tumor types.^152^, ^153^ Glioblastoma (GB) remains the most extensively studied tumor model in MBRT research. In orthotopic rat models, both X‐ray MBRT^154^, ^155^ and PMBT^7^, ^8^, ^156^, ^157^ yielded significant survival benefits. Notably, PMBT led to long‐term survival rates of up to 83%, with evidence of immunological memory in surviving animals.^13^ Other preclinical studies further support the efficacy of SFRT in various tumor types. Antitumor effects of GRID were demonstrated in a murine sarcoma model,^158^ and improved tumor control was reported in a rat orthotopic breast cancer model.^159^ In lung cancer, LRT significantly delayed tumor growth in a mouse model.^45^

#### 5.1.1 Homogeneous Irradiation

In this approach, a homogeneous lateral dose profile at the depth of the tumor is achieved.^160^ This method offers the advantage of full homogeneous dose coverage of the target while sparing proximal healthy tissue due to the spatial modulation at the entrance. This can be used for deep seated tumors with distal organs at risk (OAR), like brain tumors.^161^

Since the target dose becomes homogeneous, standard treatment planning and prescription practices can be used, including conventional dosimetric tools like dose‐volume histograms (DVHs) and conformity indices. OARs along the entrance path may benefit from reduced dose burden, potentially allowing for relaxed OAR constraints.^75^

#### 5.1.2 Heterogeneous Irradiation

In heterogeneous irradiation, the spatially fractionated pattern throughout the entire beam path is maintained, including the target volume itself. As a result, the tumor receives a non‐uniform dose, peak‐and‐valley doses do not (fully) merge.^162^

This approach explores the potential to maximize normal tissue sparing and to exploit new radiobiological effects of spatial modulation within the tumor. This can be used in superficial^77^ or extremity tumors (E.g. bone metastasis in analogy to FLASH trial^163^) with a shoot through approach. However, the non‐uniform dose distribution requires new prescription strategies both for tumors and for nearby OARs. Traditional dosimetric tools may be insufficient; instead, biologically informed metrics and dedicated optimization of peak and valley doses are necessary to ensure effective and safe treatment planning.

### 5.2 How to prescribe and plan

#### 5.2.1 Fractionation schemes

A recent survey of current SFRT clinical practices for irradiation of bulky (advanced) tumors showed that the most commonly used fractionation schemes among the participants depended on the treatment intention: (i) palliative: 1 fraction with 15‐18 Gy, (ii) curative: 1 fraction of 15‐20 Gy or 24 Gy in 3 fractions. It also showed that SFRT is mostly combined with conventional external RT, with a time gap that varied among the participants (between 1 and more than 3 days).^164^

Among other factors, the fractionation scheme will depend on (a) tumor type, size and location, (b) radiobiological response of cancerous and healthy tissues (c) beam width and spacing (spatial fractionation geometry), (d) PVDR.

It has been proposed by several studies that SFRT is integrated as a “boost”, either prior to, or after a conventional course of RT (IMRT/VMAT) or combined chemo‐external RT treatment.^165^, ^166^, ^167^, ^168^, ^169^ The addition of immunotherapy will play a significant role in elucidating optimal RT fractionation schemes that involve the use of SFRT techniques, fundamentally in deciding the most effective time interval between the two modalities.^105^, ^170^

#### 5.2.2 Treatment planning development for PMBT

To standardize the delivery and evaluation of outcomes in SFRT and more specifically PMBT, it is essential to involve developers of RT TPS. For techniques such as GRID and VMAT Lattice, the work by Grams et al. demonstrates that a comprehensive planning and evaluation protocol can be established. Moreover, certain TPS platforms (e.g. Eclipse (Varian, a Siemens Healthineers company)) already offer the necessary tools to generate reliable treatment plans for GRID and LRT, which can be effectively assessed and subjected to rigorous QA procedures.^171^

A more complex challenge lies in the development of planning tools for preclinical research, particularly when using minibeams with protons or X‐rays. In these applications, TPSs must be capable of performing dose calculations within voxelated geometries that resolve dose deposition at the scale of the beam size. MC‐based TPS implementations for medium‐energy X‐ray dose calculations, such as Muriplan, SmART‐XPS, and SmaART‐RAD, are already in use for preclinical studies involving conventional and image‐guided small animal irradiation platforms. Additionally, systems like μ‐RayStation (RaySearch Laboratories, Stockholm, Sweden) already support dose calculations for both X‐ray and proton‐based deliveries. The next step in advancing this field is the integration of mini‐beam collimators, along with precise commissioning and optimization tools that enable accurate planning with complex spatial dose distributions.

### 5.3 Recommendations

#### 5.3.1 Guidelines for PMBT prescription and assessment

Clinical and physics guidelines for PMBT should be developed in parallel to ensure safe and effective implementation. From the clinical side, treatment planning should initially respect established OAR constraints and incorporate additional considerations specific to spatially modulated dose distributions as clinical experience grows. Prins et al. demonstrates that robust clinical treatment planning of pMBRT can be established, with trade‐offs that should be considered during planning.^172^ On the physics side, guidelines should address beam quality, spatial modulation stability, and verification procedures. Evaluation criteria should include both standard clinical metrics (e.g. dose‐volume parameters, tumor coverage, OAR sparing) and parameters specific to minibeam delivery and QA (e.g. PVDR and beam alignment). Integrating these aspects will be essential for standardized treatment planning and outcome assessment within or across institutions.

As we consider translating PMBT toward clinical application, there is a critical need to clarify how treatment should be prescribed and assessed. Traditional prescription models may not fully capture the spatially modulated nature of PMBT dose distributions. Guidelines are recommended to define appropriate metrics, and how these should be reported (as discussed in Section 3.4).

Additionally, standardized methods for assessing treatment efficacy and toxicity, both in preclinical and clinical settings, are essential to enable consistent evaluation across studies and institutions. Such consensus will lay the foundations for clinical protocol development and regulatory acceptance across institutions.

#### 5.3.2 Patient sites

The selection of clinical sites for MBRT is driven partly by geometry, with bulky (≥ 5 cm in any dimension^171^) or large (≥100 cm^3^, tumors being identified as potential candidates, along with radio‐resistant tumors.^171^

Patient anatomical sites for PMBT should be selected based on both clinical relevance and geometric feasibility. Potential clinical indications include brain cancers, head and neck cancers, breast cancer (particularly posteroanterior configurations), lung tumors, and metastatic lesions, especially in anatomically favorable regions. The spatial modulation of minibeams may also offer benefit in palliative settings, where reducing toxicity to surrounding tissue is critical. Site selection should consider not only the tumor type but also beam access, motion management, and the ability to preserve minibeam geometry throughout treatment.

#### 5.3.3 Selection and standardization of endpoints

Endpoint selection for PMBT trials should be guided by consensus between radiation oncologists and medical physicists and should rely on validated and standardized criteria that are already widely used in clinical research. This ensures consistency across institutions and allows meaningful comparison with conventional treatments. Recommended endpoints include site‐specific toxicities to assess safety, and tumor control to evaluate treatment efficacy. For example, skin toxicity may be particularly relevant depending on the treatment site, and neurological toxicities may include any signs of radiation‐induced cognitive deficits, edema, or tissue necrosis.^9^

Standardized criteria for assessing both tumor control and toxicity are crucial for the reproducibility of PMBT studies. Consistency in reporting can be achieved through validated grading systems for toxicity (e.g., CTCAE) and objective imaging assessments for tumor response (e.g., RECIST 1.1^173^ or RANO criteria^174^ on e.g. MRI or CT scans). Establishing such criteria will not only harmonize treatment protocols but also improve comparability of results across institutions and clinical settings.

The authors are unaware of any ongoing PMBT clinical trials at the time of submitting this roadmap, it is perceived that initial reports may primarily describe acute toxicity and early tumor response. However, as with other oncological treatments, the ultimate evaluation of its clinical usefulness will depend on long‐term endpoints such as overall survival, patient‐reported outcomes, and quality of life, metrics that are often most relevant to patients.

#### 5.3.4 Clinical trials

For the clinical translation of PMBT, it is recommended that initial trials be conducted at a single institution to establish feasibility, safety, and workflow integration. Once these foundations are validated, multi‐institutional studies should follow to assess reproducibility and broader clinical applicability. Consistent reporting of treatment parameters, including beam characteristics, dosimetry metrics (e.g., Gy or alternative units), and spatial modulation patterns, is essential for meaningful interpretation and comparison across studies. While standardized and accurate dosimetry is not a strict requirement to initiate early‐phase trials, multi‐institutional dosimetry audits and centralized, prospective RT QA will be essential as studies expand, ensuring standardization of treatment for each patient. To establish the superiority of PMBT over standard of care, the community must systematically gather high‐quality data through clinical trials with a comparative component and validated endpoints. Extended registries, collecting data on all treated patients will further support the assessment of long‐term outcomes, including survival, late toxicity, and rare side effects.

## 6 SUMMARY OF THE ROADMAP

Preclinical studies and clinical investigations are crucial for identifying the optimal combinations, dosing regimens, for timely integration of PMBT into clinical practice. In addition, the assessment of PMBT combined with chemotherapy, immunotherapy, or other targeted agents needs to be evaluated. Such multimodal approaches may overcome current limitations of conventional radiotherapy and further improve patient outcomes in resistant and aggressive tumors compared to PMBT alone. Table 2 outlines the key milestones identified by the radiobiology, dosimetry, engineering, and clinical groups as the priority actions, building on the research that has been outlined previously in each respective section. As many of these will occur concurrently, progress in one area will inevitably inform the others.

**TABLE 2:** Milestones for PMBT implementation. Stage 1 investigates and defines the technical challenges for each respective group. Stage 2 considers feasibility and initiates early validation. Stage 3 develops mechanistic understanding and establishes frameworks for clinical implementation. Stage 4 advances biological understanding while integrating and implementing prototypes, workflows and procedures. Stage 5 focuses on standardization, optimization and validation. Stage 6 encompasses translation, scaling and deployment of all the milestones to enable routine clinical use.

	Stage					
	1	2	3	4	5	6
Preclinical	Establish key biological questions and early evidence needs	Demonstrate efficacy and normal‑tissue response	Define mechanistic pathways (cellular, tissue, systemic)	Characterize immune and vascular contributions	Link biological effects to dosimetry & modality	Validate systemic responses and integrate into translation
Dosimetry	Definition of a complete list of dosimetry parameters that should be reported	Establishment of a list of candidate reference conditions depending on application and identify preferred equipment to ensure traceability for each	Establish link between candidate reference conditions and relative dosimetry and identify a list of preferred equipment for relative dosimetry	Disseminate these recommendations for testing in PMBT facilities	Report findings for a representative set of PMBT scenarios	Make generic recommendations for reference and relative dosimetry
Engineering	Define PMBT beam‑delivery requirements (beam size, dose rate, field size, secondary radiation) and map them onto existing gantry /room constraints	Perform optics and MC simulations for magnetic focusing and collimation; identify feasible magnet systems, collimator designs, activation, and shielding needs	Build and commission research beamlines and prototype hardware (e.g. focused minibeam lines, modular multi‑slit collimators) with dedicated dosimetry and beam‑monitoring.	Integrate prototypes into TPS and QA procedures. Implement realistic beam models, define commissioning workflows, and develop QA and interlock strategies for time structure and single‑shot delivery	Standardize near‑term clinical solutions, prioritizing collimation: modular collimator designs, common support systems, harmonized dosimetry setups, and activation/staff‑safety protocols	Enable multi‑center deployment: disseminate standardized collimation solutions and, in parallel, mature high‑gradient magnetic focusing beamlines toward clinically robust, regulated systems
Clinical	Establish the clinical rationale	Build consensus on early clinical trial design	Define prescription models and dosimetry requirements	Standardize planning and QA for clinical use	Ensure regulatory and ethical preparedness	Establish collaborative structures

### 6.1 Interdisciplinary training and communication

To provide the evidence needed for clinical translation, the community must, where possible, try to reach a consensus on the terminology used in reporting. Issues in reporting are not novel and have been extensively reported in conventional radiation therapy, where a consensus on reporting was not always present.^175^ Here however, we have the added complexity of varying radiation type, fractionation pattern with varying levels of heterogeneity in dose delivery, dose rate and delivery schedule.

As with many other forms of RT, cross‐disciplinary collaboration is a cornerstone of PMBT. Any novel medical treatment is fundamentally clinically driven and optimized to try and provide the best outcomes, to the most patients, while minimizing the side effects.

However, the “why” and “what” are, at present, more important than the “how”: why does the healthy tissue toxicity decrease when we deliver PMBT, without a reduction in tumor control, and what is the optimal configuration to achieve this? Addressing these questions effectively requires a continuous dialogue between experts in beam delivery, dosimetric measurement, radiation biology and clinical treatment. Without understanding how the biological effectiveness of spatial fractionation can be optimized, highly accurate dosimetry may be wasted in measuring the wrong quantities in the wrong locations. In addition, establishing a universally agreed set of standard tests is essential to allow comparison between different spatial fractionation schemes so that effectiveness can be assessed by multiple centers in relation to a common reference. Even with a quantity as apparently straightforward as dose, differences in definition between physicists and biologists can impede communication, understanding and progress. Prezado et al.^15^ proposed that collaborative efforts focused on retrospective data analysis could help identify the most meaningful correlations between different evaluation metrics, thereby supporting the development of more appropriate prescription guidelines and improving the assessment of PMBT efficacy.

To support clinical translation, dedicated focus groups should also be established around key technical and clinical domains. For example, a dosimetry‐focused group may define recommended protocols for reference dose measurements, evaluate suitable detector types, and address challenges such as spatial resolution, energy dependence, and calibration standards for minibeam fields. Similar groups could be formed for imaging, treatment planning, quality assurance procedures, and clinical endpoint definitions, ensuring that each aspect of the workflow is addressed through common consensus and shared experience across institutions.

### 6.2 Vendor engagement

It is clear that accurate and effective dosimetry is required and ideally should be available ahead of clinical and technological drivers. To a certain extent, this can only be possible if, as discussed in Section 3, the parameters to be measured have been confirmed. In the short term, dosimetry will likely be achieved by optimizing and consolidating existing technologies whilst the field of PMBT continues to evolve. In the longer term, however, it is highly likely new methods, and equipment will be required to meet the changing needs of users as PMBT matures.

The route to market for new technologies is both costly and time‐consuming, so it is vital to engage vendors at an early stage of development. Progression through the Technology Readiness Levels, in compliance with statutory legal and safety requirements, often resulting in Medical Device Certification across several countries can be an extremely costly and time‐consuming process. Progression of preclinical work and early clinical trials may provide greater leverage for development of new technologies. However, vendors may be reluctant to develop and progress potentially high‐risk, new technologies unless there is clear evidence of a strong scientific demand and typically a world‐wide market need.

Two recent examples in adjacent radiotherapy modalities illustrate how industry engagement supports developments: the long‑standing MRI‑Linac Consortium, which has supported the establishment of MRI‑guided radiotherapy over more than a decade with Elekta's Unity system as the principal commercial platform, and early‑stage collaborations such as those between Standard Imaging and FLASH radiotherapy research groups, demonstrating how vendor involvement can help mature novel technologies when there is clear scientific momentum.

### 6.3 Evaluating and reporting PMBT plans—New ICRU recommendations

PMBT presents a challenge when applying ICRU recommendations, as its inherently non‐uniform dose distribution conflicts with the conventional assumptions of homogeneity and standardized dose reporting used in traditional RT.

One of the main difficulties with the clinical implementation of PMBT is caused by the lack of standardization on dose prescription and tools for evaluating the biological effect of the physical dose distributions delivered. That is especially enhanced when the spatially fractionated pattern is maintained along the entire beam path, down to the target volume, passing through the healthy tissue.^176^ This is to be expected, as the correlation between quantifiable dosimetric parameters and clinical treatment response in PMBT are not yet fully understood.

Recent recommendations on this topic, aiming to support clinical implementation of SFRT through the development of clinical trials are being laid out by the NRG Oncology‐AAPM working group.^105^ Groups have outlined comprehensive evaluations of the different metrics and treatment prescription approaches currently used as well as suggesting pathways to narrow the current knowledge gaps.^15^, ^177^

### 6.4 Ongoing challenges

Nevertheless, the clinical implementation of PMBT still faces challenges. First, there is the dual‐side common challenge of the translation of preclinical results to human care. Preclinical studies, especially in RT, are necessary to establish the proof of concept of an innovation, to gather initial positive comparative results and safety data, to dig deeper into underlying biomechanisms and determine the most favorable conditions of use. However, they also exhibit harsh limitations, such as the imperfection of their methods and statistics, lack of correlation between human and animal physiology, and the poorly representative surrogates of human outcomes (e.g., skin toxicity, cognitive impairment after whole brain RT and tumor growth).^178^ Preclinical models must increasingly mirror clinical scenarios. This includes the use of orthotopic rather than ectopic tumors and integrating both animal and human‐derived data, ideally using patient‐derived cells.^179^ Applying current standards of care in preclinical studies will also help evaluate how PMBT can be incorporated into existing treatment regimens, or in some cases, replace them. For instance, standard‐of‐care protocols for GB have been established in rodents^180^ and may serve as a foundation for PMBT approaches.

In addition, there needs to be a clear way to correlate radiobiological findings with clinically relevant information, where radiobiologically driven plan evaluation tools have been proposed. In the context of SFRT combined with conventional RT, biological effective dose (BED) is found to be unsuitable for spatially fractionated modalities due to the heterogeneous dose distribution and biological response. A unifying spatial BED (s‐BED) parameter has been proposed to better predict tumor and OAR response.^176^ Similarly, spatially equivalent uniform dose (s‐EUD), a variation upon the conventional equivalent uniform dose (EUD) parameter, has been proposed to represent the uniform dose that, delivered over the same number of fractions as a non‐uniform distribution, would result in the same radiobiological effect.^181^, ^182^ However, both s‐BED and s‐EUD require a robust understanding of the underlying radiobiological response prior to clinical implementation. Mathematical models incorporating radiation‐induced intercellular signaling (bystander effect) have also been developed to account for non‐localized biological responses that cannot be explained through direct dose deposition alone.^151^, ^183^, ^184^

As conventional treatments such as surgery, chemotherapy, and radiotherapy may induce radioresistance in residual tumor cells,^185^, ^186^, ^187^ assessing PMBT in these therapeutic contexts could further clarify its potential as an adjuvant or standalone therapy. Moreover, in the case of preclinical PMBT, most data were compiled using a one‐session treatment and in an entire organ while most RT schemes in human treatment are fractionated and localized.^188^ One may object that these well‐known limitations plead in favor of a rapid move toward assessing the innovation in clinical trials once reproducible high‐quality preclinical results are collected. Nevertheless, the community has identified two main gaps that must be addressed to fully realize the potential of PMBT: the limited understanding of the underlying radiobiological mechanisms, and the relationship between PMBT's dosimetric and geometric parameters and the response of both normal and tumoral tissue.^15^ As outlined in this review, the parameters of PMBT treatment are numerous and may largely vary. A key obstacle that may hinder clinical implementation is the constrained access to particle therapy worldwide. While the number of proton therapy centers is rapidly growing, with a few carbon ion facilities being planned or under construction,^189^ most patients with theoretical indications of particle therapy are still treated with photon beam therapy, as the number of available slots for particle therapy are restricted.^190^ Therefore, the initial indications for PMBT and the design of early clinical trials must be carefully chosen to avoid the dual risks of limited efficacy and excessive toxicity, which could hinder PMBT from being recognized as a valuable innovation. To prove the benefit of PMBT, clinical investigations must be carried out to produce, collect, analyze and evaluate clinical data to verify the safety, performance, and favorable benefit/risk ratio of the innovation. The innovation of PMBT falls under the regulatory process of medical device.^191^ Before a clinical trial involving a new device may begin, the proposed study and device must undergo a review and notification process to ensure the safety of study participants. This process is overseen by national or supra‐national health authorities (e.g. European medical device regulation 2017/745/EU^192^ ^)^ and requires time and skills to consider in the roadmap of the innovation's implementation.^193^ In addition, external dosimetry audits are needed to independently quantify PMBT dose delivery and confirm adherence to trial protocols,^194^ however this requires agreed upon dosimetric protocols and regulatory alignment.^195^

## 7 CONCLUSION

The resolute pace of innovation in medicine prompts significant research to gather robust and rigorous clinical data on PMBT. There remain a significant number of unanswered questions relating to the optimal form of enabling routine clinical use of collimated proton minibeams within the next five years, with other ions not long after. This roadmap summarizes the key unanswered questions and obstacles that must be addressed to enable routine clinical implementation.

## CONFLICT OF INTEREST STATEMENT

The authors declare that there is no conflict of interest.

## References

[1] Laissue JA , Blattmann H , Slatkin DN . Alban Köhler (1874‐1947): erfinder der Gittertherapie. Z Med Phys. 2012;22(2):90‐99. doi:10.1016/J.ZEMEDI.2011.07.002 21862299

[2] Theorie einer KöhlerA . Methode, bisher unmöglich unanwendbar hohe Dosen Röntgenstrahlen in der Tiefe des Gewebes zur therapeutischen Wirksamkeit zu bringen ohne schwere Schädigung des Patienten, zugleich eine Methode des Schutzes gegen Röntgenverbrennung überhaupt. Fortschr Geb Roentgenstr. 1909;14:27‐29.

[3] Curtis HJ . The use of deuteron microbeam for simulating the biological effects of heavy cosmic‐ray particles. Radiat Res Suppl. 1967;7:250‐257. doi:10.2307/3583718 6058661

[4] Yan W , Khan MK , Wu X , et al. Spatially fractionated radiation therapy: history, present and the future. Clin Transl Radiat Oncol. 2020;20:30‐38. doi:10.1016/J.CTRO.2019.10.004 31768424 PMC6872856

[5] Mayr NA , Snider JW , Regine WF , et al. An International Consensus on the Design of Prospective Clinical–Translational Trials in Spatially Fractionated Radiation Therapy. Adv Radiat Oncol. 2022;7(2):100866. doi:10.1016/J.ADRO.2021.100866 35198833 PMC8843999

[6] Prezado Y , Fois GR . Proton‐minibeam radiation therapy: a proof of concept. Med Phys. 2013;40(3):031712. doi:10.1118/1.4791648 23464307

[7] Prezado Y , Jouvion G , Patriarca A , et al. Proton minibeam radiation therapy widens the therapeutic index for high‐grade gliomas. Sci Rep. 2018;8(1):1‐10. doi:10.1038/S41598-018-34796-8 30405188 PMC6220274

[8] Prezado Y , Jouvion G , Guardiola C , et al. Tumor Control in RG2 Glioma‐Bearing Rats: a Comparison Between Proton Minibeam Therapy and Standard Proton Therapy. Int J Radiat Oncol Biol Phys. 2019;104(2):266‐271. doi:10.1016/J.IJROBP.2019.01.080 30703513

[9] Lamirault C , Doyère V , Juchaux M , et al. Short and long‐term evaluation of the impact of proton minibeam radiation therapy on motor, emotional and cognitive functions. Sci Rep. 2020;10(1):1‐14. doi:10.1038/S41598-020-70371-W 32782370 PMC7419511

[10] Prezado Y , Jouvion G , Hardy D , et al. Proton minibeam radiation therapy spares normal rat brain: long‐Term Clinical, Radiological and Histopathological Analysis. Sci Rep. 2017;7(1):1‐7. doi:10.1038/S41598-017-14786-Y;TECHMETA 29089533 PMC5663851

[11] Zlobinskaya O , Girst S , Greubel C , et al. Reduced side effects by proton microchannel radiotherapy: study in a human skin model. Radiat Environ Biophys. 2013;52(1):123‐133. doi:10.1007/S00411-012-0450-9/METRICS 23271171

[12] Bertho A , Ortiz R , Juchaux M , et al. First evaluation of temporal and spatial fractionation in proton minibeam radiation therapy of glioma‐bearing rats. Cancers (Basel). 2021;13(19):4865. doi:10.3390/CANCERS13194865/S1 34638352 PMC8507607

[13] Bertho A , Iturri L , Brisebard E , et al. Evaluation of the Role of the Immune System Response After Minibeam Radiation Therapy. Int J Radiat Oncol Biol Phys. 2023;115(2):426‐439. doi:10.1016/J.IJROBP.2022.08.011 35985455

[14] Iturri L , Riquelme‐Perez M , Bonté PE , et al. Evaluation of Proton Minibeam Radiotherapy on Antitumor Immune Responses in a Rat Model of Glioblastoma. Cancer Immunol Res. 2025;OF1‐OF19:1854‐1872. doi:10.1158/2326-6066.CIR-24-0902. Published online August 20. PMC12580782 40833465

[15] Prezado Y , Grams M , Jouglar E , et al. Spatially fractionated radiation therapy: a critical review on current status of clinical and preclinical studies and knowledge gaps. Phys Med Biol. 2024;69(10):10TR02. doi:10.1088/1361-6560/AD4192 38648789

[16] Jouglar E , de Marzi L , Verrelle P , et al. From pre‐clinical studies to human treatment with proton‐minibeam radiation therapy: adapted Idea, Development, Exploration, Assessment and Long‐term evaluation (IDEAL) framework for innovation in radiotherapy. Clin Transl Radiat Oncol. 2025;52:100932. doi:10.1016/J.CTRO.2025.100932 40124645 PMC11928333

[17] Milano MT , Marks LB , Olch AJ , et al. Comparison of Risks of Late Effects From Radiation Therapy in Children Versus Adults: insights From the QUANTEC, HyTEC, and PENTEC Efforts. Int J Radiat Oncol Biol Phys. 2024;119(2):387‐400. doi:10.1016/J.IJROBP.2023.08.066 38069917

[18] Andratschke N , Willmann J , Appelt AL , et al. European Society for Radiotherapy and Oncology and European Organisation for Research and Treatment of Cancer consensus on re‐irradiation: definition, reporting, and clinical decision making. Lancet Oncol. 2022;23(10):e469‐e478. doi:10.1016/S1470-2045(22)00447-8 36174633

[19] Prezado Y , Lamirault C , Jouglar E , et al. Proton minibeam radiation therapy: a promising alternative for brain re‐irradiations. Radiotherapy and Oncology. 2025;209:110980. doi:10.1016/j.radonc.2025.110980 40472998

[20] Song HN , Jin H , Kim JH , et al. Abscopal Effect of Radiotherapy Enhanced with Immune Checkpoint Inhibitors of Triple Negative Breast Cancer in 4T1 Mammary Carcinoma Model. Int J Mol Sci. 2021;22(19):10476. doi:10.3390/IJMS221910476 34638817 PMC8509046

[21] Jenkins SV , Johnsrud AJ , Dings RPM , Griffin RJ . Bystander effects in spatially fractionated radiation therapy: from molecule to organism to clinical implications. Semin Radiat Oncol. 2024;34(3):284. doi:10.1016/J.SEMRADONC.2024.05.004 38880537 PMC11185274

[22] Rivera JN , Laemont K , Tovmasyan A , et al. Minibeam Spatially‐Fractionated Radiation Therapy Is Superior to Uniform Dose Radiation Therapy for Abscopal Effect When Combined with PD‐L1 Checkpoint Inhibitor Immunotherapy in a Dual Tumor Murine Mammary Carcinoma Model. Radiation. 2025;5(1):3. doi:10.3390/RADIATION5010003

[23] https://www.linkedin.com/groups/9092474

[24] Zeman W , Curtis HJ , Baker CP . Histopathologic Effect of High‐Energy‐Particle Microbeams on the Visual Cortex of the Mouse Brain. Radiat Res. 1961;15(4):496‐514. doi:10.2307/3571293 14010109

[25] Prezado Y , Deman P , Varlet P , et al. Tolerance to Dose Escalation in Minibeam Radiation Therapy Applied to Normal Rat Brain: long‐Term Clinical. Radiological and Histopathological Analysis. 2015;184(3):314‐321. doi:10.1667/RR14018.1 26284420

[26] Dilmanian FA , Rusek A , Fois GR , et al. Interleaved Carbon Minibeams: an Experimental Radiosurgery Method With Clinical Potential. Int J Radiat Oncol Biol Phys. 2012;84(2):514‐519. doi:10.1016/J.IJROBP.2011.12.025 22342299

[27] Kawashiro S , Yamada S , Okamoto M , et al. Dose assessment in moving targets and organs at risk during carbon ion therapy for pancreatic cancer with respiratory gating. Phys Imaging Radiat Oncol. 2025;34(5):100775. doi:10.1016/j.ijrobp.2018.04.057 40487727 PMC12141065

[28] Bertho A , Graeff C , Ortiz R , et al. Carbon minibeam radiation therapy results in tumor growth delay in an osteosarcoma murine model. Scientific Reports. 2025;15(1):7305. doi:10.1038/s41598-025-91872-6 40025099 PMC11873225

[29] Prezado Y , Hirayama R , Matsufuji N , et al. A Potential Renewed Use of Very Heavy Ions for Therapy: neon Minibeam Radiation Therapy. Cancers. 2021;13(6):1356. doi:10.3390/CANCERS13061356 33802835 PMC8002595

[30] Prezado Y , Dos Santos M, Gonzalez W , et al. Transfer of Minibeam Radiation Therapy into a cost‐effective equipment for radiobiological studies: a proof of concept. Sci Rep. 2017;7(1):1‐10. doi:10.1038/S41598-017-17543-3 29229965 PMC5725561

[31] Fukunaga H , Kaminaga K , Sato T , et al. Spatially Fractionated Microbeam Analysis of Tissue‐sparing Effect for Spermatogenesis, 2020;194(6):698‐706. doi:10.1667/RADE-19-00018.1 33348374

[32] Sabatasso S , Laissue JA , Hlushchuk R , et al. Microbeam Radiation‐Induced Tissue Damage Depends on the Stage of Vascular Maturation. Int J Radiat Oncol Biol Phys. 2011;80(5):1522‐1532. doi:10.1016/J.IJROBP.2011.03.018 21740994

[33] Bouchet A , Serduc R , Laissue JA , Djonov V . Effects of microbeam radiation therapy on normal and tumoral blood vessels. Physica Medica. 2015;31(6):634‐641. doi:10.1016/J.EJMP.2015.04.014 26004351

[34] Bouchet A , Lemasson B , Le Duc G , et al. Preferential Effect of Synchrotron Microbeam Radiation Therapy on Intracerebral 9L Gliosarcoma Vascular Networks. Int J Radiat Oncol Biol Phys. 2010;78(5):1503‐1512. doi:10.1016/J.IJROBP.2010.06.021 20932689

[35] Fazzari J , Fernandez‐Palomo C , Pellicioli P , et al. Spatially fractionated minibeam radiation delivered at clinically feasible dose rates induces transient vascular permeability. Sci Rep. 2025;15(1):1‐14. doi:10.1038/S41598-025-87395-9 40064939 PMC11894116

[36] Asur R , Butterworth KT , Penagaricano JA , Prise KM , Griffin RJ . High dose bystander effects in spatially fractionated radiation therapy. Cancer Lett. 2015;356(1):52‐57. doi:10.1016/J.CANLET.2013.10.032 24246848 PMC4022709

[37] Asur RS , Sharma S , Chang CW , et al. Spatially Fractionated Radiation Induces Cytotoxicity and Changes in Gene Expression in Bystander and Radiation Adjacent Murine Carcinoma Cells. 2012;177(6):751‐765. doi:10.1667/RR2780.1 PMC3395590 22559204

[38] Bouchet A , Sakakini N , El Atifi M , et al. Early Gene Expression Analysis in 9L Orthotopic Tumor‐Bearing Rats Identifies Immune Modulation in Molecular Response to Synchrotron Microbeam Radiation Therapy. PLoS One. 2013;8(12):e81874. doi:10.1371/JOURNAL.PONE.0081874 24391709 PMC3876987

[39] Potez M , Fernandez‐Palomo C , Bouchet A , et al. Synchrotron Microbeam Radiation Therapy as a New Approach for the Treatment of Radioresistant Melanoma: potential Underlying Mechanisms. Int J Radiat Oncol Biol Phys. 2019;105(5):1126‐1136. doi:10.1016/J.IJROBP.2019.08.027 31461675

[40] Bazyar S , O'brien ET , Benefield T , et al. Immune‐mediated effects of microplanar radiotherapy with a small animal irradiator. Cancers (Basel). 2022;14(1):155. doi:10.3390/CANCERS14010155/S1 PMC8750301 35008319

[41] Dilmanian FA , Button TM , Le Duc G , et al. Response of rat intracranial 9L gliosarcoma to microbeam radiation therapy. Neuro Oncol. 2002;4(1):26. doi:10.1215/15228517-4-1-26 11772430 PMC1920629

[42] Dal Bello R, Becher T , Fuss MC , Krämer M , Seco J . Proposal of a Chemical Mechanism for Mini‐Beam and Micro‐Beam Efficacy. Front Phys. 2020;8:564836. doi:10.3389/FPHY.2020.564836

[43] Masilela TAM , Prezado Y . Monte Carlo study of the free radical yields in minibeam radiation therapy. Med Phys. 2023;50(8):5115‐5134. doi:10.1002/MP.16475 37211907

[44] Bertho A , Iturri L , Prezado Y . Radiation‐induced immune response in novel radiotherapy approaches FLASH and spatially fractionated radiotherapies. Int Rev Cell Mol Biol. 2023;376:37‐68. doi:10.1016/BS.IRCMB.2022.11.005 36997269

[45] Kanagavelu S , Gupta S , Wu X , et al. In Vivo Effects of Lattice Radiation Therapy on Local and Distant Lung Cancer: potential Role of Immunomodulation. Radiat Res. 2014;182(2):149‐162. doi:10.1667/RR3819.1 25036982 PMC7670883

[46] Johnsrud AJ , Jenkins SV , Jamshidi‐Parsian A , et al. Evidence for Early Stage Anti‐Tumor Immunity Elicited by Spatially Fractionated Radiotherapy‐Immunotherapy Combinations. Radiat Res. 2020;194(6):688‐697. doi:10.1667/RADE-20-00065.1 33348372 PMC8008989

[47] Folkman J . Tumor angiogenesis: therapeutic implications. New England Journal of Medicine. 1971;285:1182‐1186. 4938153 10.1056/NEJM197111182852108

[48] Baish JW , Gazit Y , Berk DA , Nozue M , Baxter LT , Jain RK . Role of Tumor Vascular Architecture in Nutrient and Drug Delivery: an Invasion Percolation‐Based Network Model. Microvasc Res. 1996;51(3):327‐346. doi:10.1006/mvre.1996.0031 8992232

[49] Ribatti D , Nico B , Vacca A , Roncali L , Dammacco F . Endothelial cell heterogeneity and organ specificity. J Hematother Stem Cell Res. 2002;11(1):81‐90. doi:10.1089/152581602753448559 11847005

[50] Barlow KD , Sanders AM , Soker S , Ergun S , Metheny‐Barlow LJ . Pericytes on the Tumor Vasculature: jekyll or Hyde?. Cancer Microenvironment. 2012;6(1):1‐17. doi:10.1007/s12307-012-0102-2 22467426 PMC3601214

[51] Lin Q , Choyke PL , Sato N . Visualizing vasculature and its response to therapy in the tumor microenvironment. Theranostics. 2023;13(15):5223‐5246. doi:10.7150/thno.84947 37908739 PMC10614675

[52] Wijerathne H , Langston JC , Yang Q , et al. Mechanisms of radiation‐induced endothelium damage: emerging models and technologies. Radiotherapy and Oncology. 2021;158:21‐32. doi:10.1016/J.RADONC.2021.02.007 33581220 PMC8119342

[53] Langley RE , Bump EA , Quartuccio SG , Medeiros D , Braunhut SJ . Radiation‐induced apoptosis in microvascular endothelial cells. Br J Cancer. 1997;75(5):666. doi:10.1038/BJC.1997.119 9043022 PMC2063324

[54] Yamazaki T , Young KH . Effects of radiation on tumor vasculature. Mol Carcinog. 2022;61(2):165‐172. doi:10.1002/MC.23360 34644811

[55] Gaugler MH , Squiban C , Van Der Meeren A , Bertho JM , Vandamme M , Mouthon MA . Late and persistent up‐regulation of intercellular adhesion molecule‐1 (ICAM‐1) expression by ionizing radiation in human endothelial cells in vitro. Int J Radiat Biol. 1997;72(2):201‐209. doi:10.1080/095530097143428 9269313

[56] Guipaud O , Jaillet C , Clément‐Colmou K , François A , Supiot S , Milliat F . The importance of the vascular endothelial barrier in the immune‐inflammatory response induced by radiotherapy. British Journal of Radiology. 2018;91(1089). doi:10.1259/BJR.20170762 PMC6223160 29630386

[57] Mondini M , Nizard M , Tran T , et al. Synergy of radiotherapy and a cancer vaccine for the treatment of HPV‐associated head and neck cancer. Mol Cancer Ther. 2015;14(6):1336‐1345. doi:10.1158/1535-7163.MCT-14-1015 25833837

[58] Sathishkumar S , Dey S , Meigooni AS , et al. The Impact of TNF‐α Induction on Therapeutic Efficacy following High Dose Spatially Fractionated (GRID) Radiation. Technol Cancer Res Treat. 2002;1(2):141‐147. doi:10.1177/153303460200100207 12622521

[59] Smith R , Wang J , Seymour C , et al. Homogenous and Microbeam X‐Ray Radiation Induces Proteomic Changes in the Brains of Irradiated Rats and in the Brains of Nonirradiated Cage Mate Rats. Dose‐Response. 2018;16(1):1559325817750068. doi:10.1177/1559325817750068 29383012 PMC5784471

[60] Lobachevsky P , Ivashkevich A , Forrester HB , et al. Assessment and Implications of Scattered Microbeam and Broadbeam Synchrotron Radiation for Bystander Effect Studies. Radiat Res. 2015;184(6):650‐659. doi:10.1667/RR13720.1 26632855

[61] Dilmanian FA , Qu Y , Feinendegen LE , et al. Tissue‐sparing effect of x‐ray microplanar beams particularly in the CNS: is a bystander effect involved?. Exp Hematol. 2007;35(4):69‐77. doi:10.1016/J.EXPHEM.2007.01.014 17379090

[62] Chandimali N , Bak SG , Park EH , et al. Free radicals and their impact on health and antioxidant defenses: a review. Cell Death Discov. 2025;11(1):19. doi:10.1038/S41420-024-02278-8 39856066 PMC11760946

[63] Zhang T , García‐Calderón D , Molina‐Hernández M , Leitão J , Hesser J , Seco J . A theoretical study of H2O2 as the surrogate of dose in minibeam radiotherapy, with a diffusion model considering radical removal process. Med Phys. 2023;50(8):5262‐5272. doi:10.1002/MP.16570 37345373

[64] Dos Santos M , Delorme R , Salmon R , Prezado Y . Minibeam radiation therapy: a micro‐ and nano‐dosimetry Monte Carlo study. Med Phys. 2020;47(3):1379‐1390. doi:10.1002/MP.14009 31900944

[65] Potiron S , Iturri L , Lamirault C , et al. Dose Heterogeneity Reduces Radiation Oxygen Dependence in Both Tumor and Healthy Tissues: the Case of Proton Minibeam Radiation Therapy. Int J Radiat Oncol Biol Phys. 2025;123(4):1132‐1145. doi:10.1016/J.IJROBP.2025.06.3859 40582600

[66] Iturri L , Jouglar E , Gilbert C , Espenon J , Juchaux M , Prezado Y . A first evaluation of the efficacy of minibeam radiation therapy combined with an immune check point inhibitor in a model of glioma‐bearing rats. Clin Transl Radiat Oncol. 2025;51:100911. doi:10.1016/J.CTRO.2025.100911 39898330 PMC11783053

[67] Schoenfeld JD , Alexander MS , Waldron TJ , et al. Pharmacological Ascorbate as a Means of Sensitizing Cancer Cells to Radio‐Chemotherapy While Protecting Normal Tissue. Semin Radiat Oncol. 2019;29(1):25‐32. doi:10.1016/J.SEMRADONC.2018.10.006 30573181 PMC6310038

[68] Fang Z , Zhou X , Zou P , et al. Minibeam radiation therapy remodels tumor microenvironment and suppresses HIF‐1α/VEGFR axis to overcome radioresistance in triple‐negative breast cancer. npj Precision Oncology. 2025;9(1):401. doi:10.1038/s41698-025-01178-z 41413184 PMC12714760

[69] Sabatasso S , Fernandez‐Palomo C , Hlushchuk R , et al. Transient and Efficient Vascular Permeability Window for Adjuvant Drug Delivery Triggered by Microbeam Radiation. Cancers. 2021;13(9):2103. doi:10.3390/cancers13092103 33925455 PMC8123803

[70] Valceski M , Engels E , Vogel S , et al. Microbeam Radiation Therapy Bio‐Dosimetry Enhanced by Novel Radiosensitiser Combinations in the Treatment of Brain Cancer. Cancers. 2024;16(24):4231. doi:10.3390/cancers16244231 39766130 PMC11674565

[71] Prezado Y , Lamirault C , Larcher T , et al. On the significance of peak dose in normal tissue toxicity in spatially fractionated radiotherapy: the case of proton minibeam radiation therapy. Radiotherapy and Oncology. 2025;205:110769. doi:10.1016/J.RADONC.2025.110769 39947329

[72] Ortiz R , De Marzi L , Prezado Y . Preclinical dosimetry in proton minibeam radiation therapy: robustness analysis and guidelines. Med Phys. 2022;49(8):5551‐5561. doi:10.1002/MP.15780 35621386 PMC9544651

[73] Ahmed M , Beyreuther E , Gantz S , et al. Design and dosimetric characterization of a transportable proton minibeam collimation system. Front Oncol. 2024;14:1473625. doi:10.3389/FONC.2024.1473625 39741979 PMC11685229

[74] Rivera JN , Kierski TM , Kasoji SK , Abrantes AS , Dayton PA , Chang SX . Conventional dose rate spatially‐fractionated radiation therapy (SFRT) treatment response and its association with dosimetric parameters—A preclinical study in a Fischer 344 rat model. PLoS One. 2020;15(6):e0229053. doi:10.1371/JOURNAL.PONE.0229053 32569277 PMC7307781

[75] Lansonneur P , Mammar H , Nauraye C , et al. First proton minibeam radiation therapy treatment plan evaluation. Sci Rep. 2020;10(1):1‐8. doi:10.1038/S41598-020-63975-9 32341427 PMC7184593

[76] Guardiola C , De Marzi L , Prezado Y . Verification of a Monte Carlo dose calculation engine in proton minibeam radiotherapy in a passive scattering beamline for preclinical trials. British Journal of Radiology. 2020;93(1107):20190578. doi:10.1259/bjr.20190578 31868523 PMC7066976

[77] Grams MP , Mateus CQ , Mashayekhi M , et al. Minibeam Radiation Therapy Treatment (MBRT): commissioning and First Clinical Implementation. Int J Radiat Oncol Biol Phys. 2024;120(5):1423‐1434. doi:10.1016/J.IJROBP.2024.06.035 39002850

[78] Davis W , Crewson C , Alexander A , Kundapur V , Cranmer‐Sargison G . Dosimetric characterization of an accessory mounted mini‐beam collimator across clinically beam matched medical linear accelerators. Biomed Phys Eng Express. 2017;3(1):015014. doi:10.1088/2057-1976/AA586D

[79] Kim M , Hwang UJ , Park K , et al. Dose Profile Modulation of Proton Minibeam for Clinical Application. Cancers (Basel). 2022;14(12):2888. doi:10.3390/CANCERS14122888/S1 35740553 PMC9221247

[80] Cranmer‐Sargison G , Crewson C , Davis WM , Sidhu NP , Kundapur V . Medical linear accelerator mounted mini‐beam collimator: design, fabrication and dosimetric characterization. Phys Med Biol. 2015;60(17):6991. doi:10.1088/0031-9155/60/17/6991 26305166

[81] Reaz F , Sitarz MK , Traneus E , Bassler N . Parameters for proton minibeam radiotherapy using a clinical scanning beam system. Acta Oncol (Madr). 2023;62(11):1561‐1565. doi:10.1080/0284186X.2023.2266125 37837215

[82] Schneider T , De Marzi L , Patriarca A , Prezado Y . Monte Carlo Comparison of Proton and Helium‐ion Minibeam Generation Techniques. Front Phys. 2021;9:595721. doi:10.3389/fphy.2021.595721

[83] Tobias Böhlen T , Psoroulas S , Aylward JD , et al. Recording and reporting of ultra‐high dose rate “FLASH” delivery for preclinical and clinical settings. Radiotherapy and Oncology. 2024;200:110507. doi:10.1016/J.RADONC.2024.110507 39245070

[84] Bourhis J , Sozzi WJ , Jorge PG , et al. Treatment of a first patient with FLASH‐radiotherapy. Radiotherapy and Oncology. 2019;139:18‐22. doi:10.1016/J.RADONC.2019.06.019 31303340

[85] Schwarz M , Traneus E , Safai S , Kolano A , van de Water S . Treatment planning for Flash radiotherapy: general aspects and applications to proton beams. Med Phys. 2022;49(4):2861‐2874. doi:10.1002/MP.15579 35213040

[86] McGarrigle JM , Long KR , Prezado Y . On the significance of the different geometrical and dosimetric parameters in microbeam and minibeam radiation therapy a retrospective evaluation. Front Oncol. 2024;14:1449293. doi:10.3389/FONC.2024.1449293 39403332 PMC11472836

[87] Zhang H , Grams M , Foy J , Mayr N . A Dosimetric Parameter Reference Look‐Up Table for GRID Collimator‐Based Spatially Fractionated Radiation Therapy. Cancers. 2022;14(4):1037. doi:10.3390/CANCERS14041037 35205785 PMC8869958

[88] Guardiola C , Peucelle C , Prezado Y . Optimization of the mechanical collimation for minibeam generation in proton minibeam radiation therapy. Med Phys. 2017;44(4):1470‐1478. doi:10.1002/mp.12131 28129665

[89] Prado A , Martí J , García de Acilu P , et al. Dosimetrical and geometrical parameters in single‐fraction lattice radiotherapy for the treatment of bulky tumors: insights from initial clinical experience. Physica Medica. 2024;123:103408. doi:10.1016/J.EJMP.2024.103408 38889590

[90] Sammer M , Girst S , Dollinger G . Optimizing proton minibeam radiotherapy by interlacing and heterogeneous tumor dose on the basis of calculated clonogenic cell survival. Sci Rep. 2021;11(1):1‐16. doi:10.1038/S41598-021-81708-4 33574390 PMC7878903

[91] Pshenichnov IA , Dmitrieva UA , Savenkov SD , Svetlichnyi AO . Proton and Carbon‐Ion Minibeam Therapy: from Modeling to Treatment. Physics of Particles and Nuclei. 2024;55(4):929‐934. doi:10.1134/S1063779624700606

[92] Debrot E , Bolst D , James B , et al. Investigating Variable RBE in a 12C Minibeam Field with Microdosimetry and GEANT4. Radiat Prot Dosimetry. 2019;183(1‐2):160‐166. doi:10.1093/rpd/ncy234 30668821

[93] Green S , Lourenço A , Palmans H , et al. IPEM code of practice for proton therapy dosimetry based on the NPL primary standard proton calorimeter calibration service. Phys Med Biol. 2025;70(6):065016. doi:10.1088/1361-6560/adad2e 39842088

[94] INTERNATIONAL ATOMIC ENERGY AGENCY . Absorbed Dose Determination in External Beam Radiotherapy. Technical Reports Series No. 398 (Rev. 1). 2024:1‐286. doi:10.61092/IAEA.VE7Q-Y94K. Published online.

[95] Almond PR , Biggs PJ , Coursey BM , et al. AAPM's TG‐51 protocol for clinical reference dosimetry of high‐energy photon and electron beams. Med Phys. 1999;26(9):1847‐1870. doi:10.1118/1.598691 10505874

[96] Schneider T . Technical aspects of proton minibeam radiation therapy: minibeam generation and delivery. Physica Medica. 2022;100:64‐71. doi:10.1016/J.EJMP.2022.06.010 35750002

[97] Bassler N , Schettino G , Palmans H , et al. The Biologically Effective Particle Number Ratio (BPNR): a new framework to quantify the therapeutic window in SFRT and other modalities. Published online November 27, 2025. Accessed March 25, 2026. http://arxiv.org/abs/2511.22716

[98] Elbanna M , Chowdhury NN , Rhome R , Fishel ML . Clinical and Preclinical Outcomes of Combining Targeted Therapy With Radiotherapy. Front Oncol. 2021;11:749496. doi:10.3389/FONC.2021.749496 34733787 PMC8558533

[99] Palmans H , Andreo P , Huq MS , Seuntjens J , Christaki KE , Meghzifene A . Dosimetry of small static fields used in external photon beam radiotherapy: summary of TRS‐483, the IAEA–AAPM international Code of Practice for reference and relative dose determination. Med Phys. 2018;45(11):e1123‐e1145. doi:10.1002/MP.13208 30247757

[100] Cotterill J , Flynn S , Thomas R , et al. Challenges for the Implementation of Primary Standard Dosimetry in Proton Minibeam Radiation Therapy. Cancers. 2024;16(23):4013. doi:10.3390/CANCERS16234013 39682199 PMC11640736

[101] Prezado Y , Vautrin M , Martínez‐Rovira I , et al. Dosimetry protocol for the forthcoming clinical trials in synchrotron stereotactic radiation therapy (SSRT). Med Phys. 2011;38(3):1709‐1717. doi:10.1118/1.3556561 21520884

[102] Zhu XR , Poenisch F , Lii M , et al. Commissioning dose computation models for spot scanning proton beams in water for a commercially available treatment planning system. Med Phys. 2013;40(4):041723. doi:10.1118/1.4798229 23556893 PMC3631269

[103] Schwaab J , Brons S , Fieres J , Parodi K . Experimental characterization of lateral profiles of scanned proton and carbon ion pencil beams for improved beam models in ion therapy treatment planning. Phys Med Biol. 2011;56(24):7813. doi:10.1088/0031-9155/56/24/009 22112370

[104] Kretschmer J , Brodbek L , Behrends C , et al. Comprehensive investigation of lateral dose profile and output factor measurements in small proton fields from different delivery techniques. Med Phys. 2023;50(7):4546‐4561. doi:10.1002/mp.16357 36908165

[105] Li H , Mayr NA , Griffin RJ , et al. Overview and Recommendations for Prospective Multi‐institutional Spatially Fractionated Radiation Therapy Clinical Trials. Int J Radiat Oncol Biol Phys. 2024;119(3):737‐749. doi:10.1016/J.IJROBP.2023.12.013 38110104 PMC11162930

[106] Gao M , Mohiuddin MM , Hartsell WF , Pankuch M . Spatially fractionated (GRID) radiation therapy using proton pencil beam scanning (PBS): feasibility study and clinical implementation. Med Phys. 2018;45(4):1645‐1653. doi:10.1002/MP.12807 29431867

[107] Zhang H , Wu X , Zhang X , et al. Photon GRID Radiation Therapy: a Physics and Dosimetry White Paper from the Radiosurgery Society (RSS) GRID/LATTICE, Microbeam and FLASH Radiotherapy Working Group. Radiat Res. 2020;194(6):665‐677. doi:10.1667/RADE-20-00047.1 33348375

[108] Thevenet F , Keshmiri S , Degouttes J , et al. Microstrip plastic scintillating detector system for quality assurance in synchrotron microbeam radiotherapy. Sci Rep. 2025;15(1):1‐11. doi:10.1038/S41598-024-80736-0 39747355 PMC11696310

[109] Flynn S , Allport P , De Marzi L , et al. Development of CMOS dosimetry in proton minibeams for enhanced QA and primary standard absorbed dose calorimetry. Journal of Instrumentation. 2023;18(03):P03014. doi:10.1088/1748-0221/18/03/P03014

[110] Flynn S , Price T , Allport PP , et al. Evaluation of a pixelated large format CMOS sensor for x‐ray microbeam radiotherapy. Med Phys. 2020;47(3):1305‐1316. doi:10.1002/MP.13971 31837272 PMC7078942

[111] Bazyar S , Inscoe CR , O'Brian ET , Zhou O , Lee YZ . Minibeam radiotherapy with small animal irradiators; in vitro and in vivo feasibility studies. Phys Med Biol. 2017;62(23):8924. doi:10.1088/1361-6560/AA926B 29125832

[112] Sipilä P , Ojala J , Kaijaluoto S , Jokelainen I , Kosunen A . Gafchromic EBT3 film dosimetry in electron beams — energy dependence and improved film read‐out. J Appl Clin Med Phys. 2016;17(1):360‐373. doi:10.1120/JACMP.V17I1.5970 26894368 PMC5690204

[113] Casolaro P , Campajola L , Breglio G , et al. Real‐time dosimetry with radiochromic films. Sci Rep. 2019;9(1):1‐11. doi:10.1038/S41598-019-41705-0 30926839 PMC6440967

[114] Brace OJ , Alhujaili SF , Paino JR , et al. Evaluation of the PTW microDiamond in edge‐on orientation for dosimetry in small fields. J Appl Clin Med Phys. 2020;21(8):278‐288. doi:10.1002/ACM2.12906 PMC7484886 32441884

[115] Granado‐Gonzalez M , Price T , Gonella L , et al. First test beam of the DMAPS‐based proton tracker at the pMBRT facility at the Curie Institute. Phys Med Biol. 2024;69(21):215026. doi:10.1088/1361-6560/AD84B3 39378901

[116] Sotiropoulos M , Prezado Y . Radiation quality correction factors for improved dosimetry in preclinical minibeam radiotherapy. Med Phys. 2022;49(10):6716‐6727. doi:10.1002/MP.15838 35904962 PMC9804712

[117] Archer J , Li E , Petasecca M , et al. Synchrotron X‐ray microbeam dosimetry with a 20 micrometre resolution scintillator fibre‐optic dosimeter. J Synchrotron Radiat. 2018;25(Pt 3):826‐832. doi:10.1107/S1600577518003016 29714194

[118] Taylor JT , Waltham C , Price T , et al. A new silicon tracker for proton imaging and dosimetry. Nucl Instrum Methods Phys Res A. 2016;831:362‐366. doi:10.1016/J.NIMA.2016.02.013 27667884 PMC5002944

[119] Davis JA , Engels E , Petasecca M , et al. X‐TREAM protocol for in vitro microbeam radiation therapy at the Australian Synchrotron. J Appl Phys. 2021;129(24):244902. doi:10.1063/5.0040013

[120] Darafsheh A , Goddu SM , Williamson J , Zhang T , Sobotka LG . Radioluminescence Dosimetry in Modern Radiation Therapy. Adv Photonics Res. 2024;5(9):2300350. doi:10.1002/ADPR.202300350

[121] Ciarrocchi E , Ravera E , Cavalieri A , et al. Plastic scintillator‐based dosimeters for ultra‐high dose rate (UHDR) electron radiotherapy. Physica Medica. 2024;121:103360. doi:10.1016/J.EJMP.2024.103360 38692114

[122] Kunkyab T , Magliari A , Jirasek A , Mou B , Hyde D . Semi‐automated vertex placement for lattice radiotherapy and dosimetric verification using 3D polymer gel dosimetry. J Appl Clin Med Phys. 2024;25(11):e14489. doi:10.1002/ACM2.14489 39186819 PMC11540016

[123] Wang S , Gonzalez G , Sun L , et al. Real‐time tracking of the Bragg peak during proton therapy via 3D protoacoustic Imaging in a clinical scenario. npj Imaging. 2024;2(1):1‐9. doi:10.1038/S44303-024-00039-X 40078731 PMC11893450

[124] Lehrack S , Assmann W , Bertrand D , et al. Submillimeter ionoacoustic range determination for protons in water at a clinical synchrocyclotron. Phys Med Biol. 2017;62(17):L20. doi:10.1088/1361-6560/AA81F8 28742053

[125] Petrich C , Winter J , Dimroth A , et al. Commissioning, characterization and first high dose rate irradiations at a compact X‐ray tube for microbeam and minibeam radiation therapy. Int J Radiat Oncol Biol Phys. 2026;124(4):1137‐1146. doi:10.1016/J.IJROBP.2025.10.012. Published online October 22, 2025. 41135710

[126] Koehler AM , Schneider RJ , Sisterson JM . Range modulators for protons and heavy ions. Nuclear Instruments and Methods. 1975;131(3):437‐440. doi:10.1016/0029-554X(75)90430-9

[127] Schneider T , Patriarca A , Degiovanni A , Gallas M , Prezado Y . Conceptual Design of a Novel Nozzle Combined with a Clinical Proton Linac for Magnetically Focussed Minibeams. Cancers. 2021;13(18):4657. doi:10.3390/CANCERS13184657 34572884 PMC8467416

[128] Schneider T , De Marzi L , Patriarca A , Prezado Y . Advancing proton minibeam radiation therapy: magnetically focussed proton minibeams at a clinical centre. Sci Rep. 2020;10(1):1‐10. doi:10.1038/S41598-020-58052-0 31992757 PMC6987213

[129] Girst S , Greubel C , Reindl J , et al. Proton Minibeam Radiation Therapy Reduces Side Effects in an In Vivo Mouse Ear Model. Int J Radiat Oncol Biol Phys. 2016;95(1):234‐241. doi:10.1016/J.IJROBP.2015.10.020 26692028

[130] Datzmann G , Sammer M , Girst S , Mayerhofer M , Dollinger G , Reindl J . Preclinical Challenges in Proton Minibeam Radiotherapy: physics and Biomedical Aspects. Front Phys. 2020;8:568206. doi:10.3389/FPHY.2020.568206

[131] Dittwald AKDB , Upgrade plans and new target stations for the HZB cyclotron. In: *Proc. IPAC’23*. IPAC’23 ‐ 14th International Particle Accelerator Conference. JACoW Publishing, Geneva, Switzerland; 2023:5169‐5172. doi:10.18429/JACoW-IPAC2023-THPM129

[132] Rousseti ADDKDN , et al. Current status of MINIBEE: minibeam beamline for preclinical experiments on spatial fractionation in the FLASH regime. In: *Proc. IPAC’24*. IPAC’24 ‐ 15th International Particle Accelerator Conference. JACoW Publishing, Geneva, Switzerland; 2024:3663‐3666. doi:10.18429/JACoW-IPAC2024-THPR62

[133] Bartzsch S , Corde S , Crosbie JC , et al. Technical advances in x‐ray microbeam radiation therapy. Phys Med Biol. 2020;65(2):02TR01. doi:10.1088/1361-6560/AB5507 31694009

[134] Dilmanian FA , Zhong Z , Bacarian T , et al. Interlaced x‐ray microplanar beams: a radiosurgery approach with clinical potential. Proceedings of the National Academy of Sciences. 2006;103(25):9709‐9714. doi:10.1073/PNAS.0603567103 PMC1480471 16760251

[135] Volz L , Reidel CA , Durante M , et al. Investigating Slit‐Collimator‐Produced Carbon Ion Minibeams with High‐Resolution CMOS Sensors. Instruments. 2023;7(2):18. doi:10.3390/instruments7020018

[136] Bertho A , Ortiz R , Maurin M , et al. Thoracic Proton Minibeam Radiation Therapy: tissue Preservation and Survival Advantage Over Conventional Proton Therapy. Int J Radiat Oncol Biol Phys. 2024;120(2):579‐592. doi:10.1016/j.ijrobp.2024.04.011 38621606

[137] Reaz F , Traneus E , Bassler N . Commissioning of a multislit collimator system for experimental pMBRT studies with uniform target dose. Phys Med Biol. 2025;70(13):135001. doi:10.1088/1361-6560/ADE04B 40460849

[138] Beyreuther E , Baumann M , Enghardt W , et al. Research Facility for Radiobiological Studies at the University Proton Therapy Dresden. Int J Part Ther. 2018;5(1):172‐182. doi:10.14338/IJPT-18-00008.1 31773028 PMC6871594

[139] Dorman EF , Emery RC , The University of Washington Clinical Cyclotron a Summary of Current Particles and Energies Used in Therapy, Isotope Production, and Clinical Research. In: Proc. Cyclotrons’13 . JACoW Publishing, Geneva, Switzerland; 2013:454‐457. https://jacow.org/CYCLOTRONS2013/papers/TH2PB03.pdf

[140] Lin Y , Li W , Wang A , Johnson D , Gan GN , Gao H . Comprehensive dosimetric commissioning of proton minibeam radiotherapy on a single gantry proton system. Front Oncol. 2024;14:1421869. doi:10.3389/FONC.2024.1421869 39099699 PMC11294745

[141] Hecksel D , Anferov V , Fitzek M , Shahnazi K . Influence of beam efficiency through the patient‐specific collimator on secondary neutron dose equivalent in double scattering and uniform scanning modes of proton therapy. Med Phys. 2010;37(6):2910‐2917. doi:10.1118/1.3431575 20632602

[142] Winterhalter C , Meier G , Oxley D , et al. Reduction of the secondary neutron dose in passively scattered proton radiotherapy, using an optimized pre‐collimator/collimator. Phys Med Biol. 2009;54(20):6065. doi:10.1088/0031-9155/54/20/003 19779218 PMC3688272

[143] Yonai S , Matsufuji N , Kanai T . Monte Carlo study on secondary neutrons in passive carbon‐ion radiotherapy: identification of the main source and reduction in the secondary neutron dose. Med Phys. 2009;36(10):4830‐4839. doi:10.1118/1.3220624 19928113

[144] Carnicer A , Candela‐Juan C , Nirrengarten M , et al. Activation of Collimators Irradiated with Clinical Proton Beams and Development of a Semiempirical Model for Activity Calculation. Health Phys. 2019;117(5):509‐525. doi:10.1097/HP.0000000000001082 31211755

[145] Bäcker CM , Bäumer C , Gerhardt M , et al. Evaluation of the activation of brass apertures in proton therapy using gamma‐ray spectrometry and Monte Carlo simulations. Journal of Radiological Protection. 2020;40(3):848. doi:10.1088/1361-6498/AB9F42 32575092

[146] Moskvin V , Cheng CW , Das IJ . Pitfalls of tungsten multileaf collimator in proton beam therapy. Med Phys. 2011;38(12):6395‐6406. doi:10.1118/1.3658655 22149823

[147] Charyyev S , Artz M , Szalkowski G , et al. Optimization of hexagonal‐pattern minibeams for spatially fractionated radiotherapy using proton beam scanning. Med Phys. 2020;47(8):3485‐3495. doi:10.1002/MP.14192 32319098

[148] Iori F , Trojani V , Zamagni A , et al. Spatially Fractionated Radiation Therapy for Palliation in Patients With Large Cancers: a Retrospective Study. Adv Radiat Oncol. 2025;10(1):101665. doi:10.1016/J.ADRO.2024.101665 39687474 PMC11647081

[149] Iori F , Cappelli A , D'Angelo E , et al. Lattice Radiation Therapy in clinical practice: a systematic review. Clin Transl Radiat Oncol. 2023;39:100569. doi:10.1016/J.CTRO.2022.100569 36590825 PMC9800252

[150] Billena C , Khan AJ . A Current Review of Spatial Fractionation: back to the Future?. Int J Radiat Oncol Biol Phys. 2019;104(1):177‐187. doi:10.1016/J.IJROBP.2019.01.073 30684666 PMC7443362

[151] Jacobson MA , Sharifzadeh Y , Dudek AZ , et al. Minibeam Radiation Therapy for Recurrent Mucosal Melanoma: an Eye‐Opening Response. Mayo Clin Proc Innov Qual Outcomes. 2025;9(4):100640. doi:10.1016/J.MAYOCPIQO.2025.100640 40686534 PMC12271886

[152] Adam JF , Balosso J , Bayat S , et al. Toward Neuro‐Oncologic Clinical Trials of High‐Dose‐Rate Synchrotron Microbeam Radiation Therapy: first Treatment of a Spontaneous Canine Brain Tumor. Int J Radiat Oncol Biol Phys. 2022;113(5):967‐973. doi:10.1016/J.IJROBP.2022.04.022 35483539

[153] Kundapur V , Mayer M , Auer RN , et al. Is Mini Beam Ready for Human Trials? Results of Randomized Study of Treating De‐Novo Brain Tumors in Canines Using Linear Accelerator Generated Mini Beams. Radiat Res. 2022;198(2):162‐171. doi:10.1667/RADE-21-00093.1 35536992

[154] Sotiropoulos M , Brisebard E , Le Dudal M , et al. X‐rays minibeam radiation therapy at a conventional irradiator: pilot evaluation in F98‐glioma bearing rats and dose calculations in a human phantom. Clin Transl Radiat Oncol. 2021;27:44‐49. doi:10.1016/J.CTRO.2021.01.001 33511291 PMC7817429

[155] Prezado Y , Sarun S , Gil S , Deman P , Bouchet A , Le Duc G . Increase of lifespan for glioma‐bearing rats by using minibeam radiation therapy. J Synchrotron Radiat. 2012;19(Pt 1):60‐65. doi:10.1107/S0909049511047042 22186645

[156] Potiron S , Iturri L , Juchaux M , et al. The significance of dose heterogeneity on the anti‐tumor response of minibeam radiation therapy. Radiotherapy and Oncology. 2024;201:110577. doi:10.1016/J.RADONC.2024.110577 39393469

[157] Bertho A , Brisebard E , Juchaux M , et al. Anti‐Tumor Immune Response and Long‐Term Immunological Memory Induced by Minibeam Radiation Therapy: a Pilot Study. Int J Radiat Oncol Biol Phys. 2021;111(3):S121‐S122. doi:10.1016/J.IJROBP.2021.07.278

[158] Johnson TR , Bassil AM , Williams NT , et al. An investigation of kV mini‐GRID spatially fractionated radiation therapy: dosimetry and preclinical trial. Phys Med Biol. 2022;67(4):045017. doi:10.1088/1361-6560/AC508C PMC9167045 35100573

[159] Sharma S , Narayanasamy G , Przybyla B , et al. Advanced Small Animal Conformal Radiation Therapy Device. Technol Cancer Res Treat. 2017;16(1):45‐56. doi:10.1177/1533034615626011 26792490 PMC5616115

[160] Meyer J , Eley J , Schmid TE , Combs SE , Dendale R , Prezado Y . Spatially fractionated proton minibeams. British Journal of Radiology. 2019;92(1095):20180466. doi:10.1259/BJR.20180466 30359081 PMC6541186

[161] Eley JG , Chadha AS , Quini C , et al. Pilot study of neurologic toxicity in mice after proton minibeam therapy. Sci Rep. 2020;10(1):1‐9. doi:10.1038/S41598-020-68015-0 32647361 PMC7347840

[162] Prezado Y . Divide and conquer: spatially fractionated radiation therapy. Expert Rev Mol Med. 2022;24:e3. doi:10.1017/ERM.2021.34

[163] Daugherty EC , Mascia A , Zhang Y , et al. FLASH Radiotherapy for the Treatment of Symptomatic Bone Metastases (FAST‐01): protocol for the First Prospective Feasibility Study. JMIR Res Protoc. 2023;12(1):e41812. doi:10.2196/41812 36206189 PMC9893728

[164] Mayr NA , Mohiuddin M , Snider JW , et al. Practice Patterns of Spatially Fractionated Radiation Therapy: a Clinical Practice Survey. Adv Radiat Oncol. 2024;9(2):101308. doi:10.1016/J.ADRO.2023.101308 38405319 PMC10885580

[165] Potez M , Bouchet A , Flaender M , et al. Synchrotron X‐Ray Boost Delivered by Microbeam Radiation Therapy After Conventional X‐Ray Therapy Fractionated in Time Improves F98 Glioma Control. Int J Radiat Oncol Biol Phys. 2020;107(2):360‐369. doi:10.1016/J.IJROBP.2020.02.023 32088292

[166] Jaekel F , Bräuer‐Krisch E , Bartzsch S , et al. Microbeam Irradiation as a Simultaneously Integrated Boost in a Conventional Whole‐Brain Radiotherapy Protocol. International Journal of Molecular Sciences. 2022;23(15):8319. doi:10.3390/IJMS23158319 35955454 PMC9368396

[167] Amendola BE , Perez NC , Mayr NA , Wu X , Amendola M . Spatially Fractionated Radiation Therapy Using Lattice Radiation in Far‐advanced Bulky Cervical Cancer: a Clinical and Molecular Imaging and Outcome Study. Radiat Res. 2020;194(6):724‐736. doi:10.1667/RADE-20-00038.1 32853384

[168] Choi JI , Daniels J , Cohen D , et al. Clinical Outcomes of Spatially Fractionated GRID Radiotherapy in the Treatment of Bulky Tumors of the Head and Neck. Cureus. 2019;11(5):e4637. doi:10.7759/CUREUS.4637 31312563 PMC6623998

[169] Serduc R , Bouchet A . MRT‐boost as the last fraction may be the most efficient irradiation schedule for increased survival times in a rat glioma model. J Synchrotron Rad. 2023;30:591‐595. doi:10.1107/S1600577523002606 PMC10161883 37067258

[170] Lu Q , Yan W , Zhu A , Tubin S , Mourad WF , Yang J . Combining spatially fractionated radiation therapy (SFRT) and immunotherapy opens new rays of hope for enhancing therapeutic ratio. Clin Transl Radiat Oncol. 2024;44:100691. doi:10.1016/J.CTRO.2023.100691 38033759 PMC10684810

[171] Grams MP , Deufel CL , Kavanaugh JA , et al. Clinical aspects of spatially fractionated radiation therapy treatments. Physica Medica. 2023;111:102616. doi:10.1016/J.EJMP.2023.102616 37311338

[172] Prins AC , Traneus E , Hoogeman MS , Ng Wei , Siang K . Impact of center‐to‐center distance and slit width on robust target coverage and PVDR for proton minibeam radiation therapy using a commercial TPS. Med Phys. 2026;53(2):e70340. doi:10.1002/mp.70340 41665563 PMC12889204

[173] Eisenhauer EA , Therasse P , Bogaerts J , et al. New response evaluation criteria in solid tumours: revised RECIST guideline (version 1.1). Eur J Cancer. 2009;45(2):228‐247. doi:10.1016/J.EJCA.2008.10.026 19097774

[174] Leao DJ , Craig PG , Godoy LF , Leite CC , Policeni B . Response Assessment in Neuro‐Oncology Criteria for Gliomas: practical Approach Using Conventional and Advanced Techniques. American Journal of Neuroradiology. 2020;41(1):10‐20. doi:10.3174/AJNR.A6358 31857322 PMC6975322

[175] Draeger E , Sawant A , Johnstone C , et al. A Dose of Reality: how 20 Years of Incomplete Physics and Dosimetry Reporting in Radiobiology Studies May Have Contributed to the Reproducibility Crisis. Int J Radiat Oncol Biol Phys. 2020;106(2):243‐252. doi:10.1016/J.IJROBP.2019.06.2545 31288053

[176] Misa J , Knight J , Clair WS , Pokhrel D . Plan evaluation tool for spatially fractionated radiation therapy for unresectable large tumors via spatial biological effective dose modeling in combination therapy. Medical Dosimetry. 2025;50(3):237‐243. doi:10.1016/j.meddos.2025.02.002 40121113 PMC12453109

[177] Fernandez‐Palomo C , Chang S , Prezado Y . Should Peak Dose Be Used to Prescribe Spatially Fractionated Radiation Therapy?—A Review of Preclinical Studies. Cancers (Basel). 2022;14(15):3625. doi:10.3390/CANCERS14153625/S1 35892895 PMC9330631

[178] Seyhan AA . Lost in translation: the valley of death across preclinical and clinical divide – identification of problems and overcoming obstacles. Translational Medicine Communications. 2019;4(1):1‐19. doi:10.1186/S41231-019-0050-7

[179] Golebiewska A , Fields RC . Advancing preclinical cancer models to assess clinically relevant outcomes. BMC Cancer. 2023;23(1):1‐3. doi:10.1186/S12885-023-10715-7 36899363 PMC10007806

[180] Le Reste PJ , Pineau R , Voutetakis K , et al. Local intracerebral inhibition of IRE1 by MKC8866 sensitizes glioblastoma to irradiation/chemotherapy in vivo. Cancer Lett. 2020;494:73‐83. doi:10.1016/J.CANLET.2020.08.028 32882336

[181] Allen Li X, Alber M , Deasy JO , et al. The use and QA of biologically related models for treatment planning: short report of the TG‐166 of the therapy physics committee of the AAPM. Med Phys. 2012;39(3):1386‐1409. doi:10.1118/1.3685447 22380372

[182] Meyer J , Stewart RD , Smith D , et al. Biological and dosimetric characterisation of spatially fractionated proton minibeams. Phys Med Biol. 2017;62(24):9260. doi:10.1088/1361-6560/aa950c 29053105

[183] Balvasi E , Mahmoudi F , Geraily G , Farnia P , Jafari F . Modeling the impact of intercellular signaling on dose metrics and therapeutic outcomes in spatially fractionated radiation therapy (SFRT) for lung cancer. Sci Rep. 2025;15(1):1‐18. doi:10.1038/S41598-025-11937-4 40764631 PMC12325640

[184] McMahon SJ , Butterworth KT , Trainor C , et al. A Kinetic‐Based Model of Radiation‐Induced Intercellular Signalling. PLoS One. 2013;8(1):e54526. doi:10.1371/JOURNAL.PONE.0054526 23349919 PMC3551852

[185] Knudsen AM , Halle B , Cédile O , et al. Surgical resection of glioblastomas induces pleiotrophin‐mediated self‐renewal of glioblastoma stem cells in recurrent tumors. Neuro Oncol. 2021;24(7):1074. doi:10.1093/NEUONC/NOAB302 PMC9248408 34964899

[186] Aasland D , Gotzinger L , Hauck L , et al. Temozolomide induces senescence and repression of DNA repair pathways in glioblastoma cells via activation of ATR–Chk1, p21, and NF‐kB. Cancer Res. 2019;79(1):99‐113. doi:10.1158/0008-5472.CAN-18-1733 30361254

[187] Li HF , Kim JS , Waldman T . Radiation‐induced Akt activation modulates radioresistance in human glioblastoma cells. Radiation Oncology. 2009;4(1):43. doi:10.1186/1748-717X-4-43 19828040 PMC2765447

[188] Rodin D , Tawk B , Mohamad O , et al. Hypofractionated radiotherapy in the real‐world setting: an international ESTRO‐GIRO survey. Radiotherapy and Oncology. 2021;157:32‐39. doi:10.1016/J.RADONC.2021.01.003 33453312 PMC8053676

[189] PTCOG ‐ Home . Accessed December 4, 2023. https://ptcog.site/

[190] Gaito S , Aznar MC , Burnet NG , et al. Assessing Equity of Access to Proton Beam Therapy: a Literature Review. Clin Oncol. 2023;35(9):e528‐e536. doi:10.1016/J.CLON.2023.05.014 37296036

[191] Medical devices | European Medicines Agency (EMA) . Accessed September 17, 2025. https://www.ema.europa.eu/en/human‐regulatory‐overview/medical‐devices

[192] Regulation ‐ 2017/745 ‐ EN ‐ Medical Device Regulation ‐ EUR‐Lex. Accessed September 17, 2025. https://eur‐lex.europa.eu/eli/reg/2017/745/oj/eng

[193] Ma Y , Zhang T , Selvaraj B , et al. Advancing Proton FLASH Radiation Therapy: innovations, Techniques, and Clinical Potentials. Int J Radiat Oncol Biol Phys. 2025;123(3):876‐890. doi:10.1016/J.IJROBP.2025.05.076. Published online June 11, 2025. 40513679

[194] Misson S , Palmer AL , Diez P , Hussein M , Clark CH . National radiotherapy dosimetry audit in the UK – A vision and roadmap. IPEM‐Translation. 2024;12(4):100032. doi:10.1016/j.ipemt.2024.100032

[195] Clark CH , Jornet N , Muren LP . The role of dosimetry audit in achieving high quality radiotherapy. Phys Imaging Radiat Oncol. 2018;5:85. doi:10.1016/j.phro.2018.03.009 33458374 PMC7807592

